# Brain- and brain tumor-penetrating disulfiram nanoparticles: Sequence of cytotoxic events and efficacy in human glioma cell lines and intracranial xenografts

**DOI:** 10.18632/oncotarget.23320

**Published:** 2017-12-15

**Authors:** Hanumantha Rao Madala, Surendra R. Punganuru, Francis Ali-Osman, Ruiwen Zhang, Kalkunte S. Srivenugopal

**Affiliations:** ^1^ Department of Biomedical Sciences, School of Pharmacy, Texas Tech University Health Sciences Center, Amarillo, TX, USA; ^2^ Department of Surgery and the Brain Tumor Center, Duke University, Durham, NC, USA; ^3^ Department of Pharmacological and Pharmaceutical Sciences, College of Pharmacy, University of Houston, Houston, TX, USA

**Keywords:** disulfiram, MGMT, glioma, chemotherapy, nanoparticles

## Abstract

There is great interest in repurposing disulfiram (DSF), a rapidly metabolizing nontoxic drug, for brain cancers and other cancers. To overcome the instability and low therapeutic efficacy, we engineered passively-targeted DSF-nanoparticles (DSFNPs) using biodegradable monomethoxy (polyethylene glycol) d,l-lactic-co-glycolic acid (mPEG-PLGA) matrix. The physicochemical properties, cellular uptake and the blood brain-barrier permeability of DSFNPs were investigated. The DSFNPs were highly stable with a size of ∼70 nm with a >90% entrapment. Injection of the nanoparticles labeled with HITC, a near-infrared dye into normal mice and tumor-bearing nude mice followed by *in vivo* imaging showed a selective accumulation of the formulation within the brain and subcutaneous tumors for >24 h, indicating an increased plasma half-life and entry of DSF into desired sites. The DSFNPs induced a potent and preferential killing of many brain tumor cell lines in cytotoxicity assays. Confocal microscopy showed a quick internalization of the nanoparticles in tumor cells followed by initial accumulation in lysosomes and subsequently in mitochondria. DSFNPs induced high levels of ROS and led to a marked loss of mitochondrial membrane potential. Activation of the MAP-kinase pathway leading to a nuclear translocation of apoptosis-inducing factor and altered expression of apoptotic and anti-apoptotic proteins were also observed. DSFNPs induced a powerful and significant regression of intracranial medulloblastoma xenografts compared to the marginal efficacy of unencapsulated DSF. Together, we show that passively targeted DSFNPs can affect multiple targets, trigger potent anticancer effects, and can offer a sustained drug supply for brain cancer treatment through an enhanced permeability retention (EPR).

## INTRODUCTION

Disulfiram (DSF, Antabuse) was the first drug approved by the U.S. Food and Drug Administration to treat chronic alcohol dependence [1]. DSF (tetraethylthiuram disulfiram) is a hydrophobic, symmetrical molecule of the dithiocarbamate family and is available in oral tablets. The drug has a strong affinity for protein-bound and unbound thiols and forms a covalent linkage with an active-site cysteine of the aldehyde dehydrogenase (ALDH) to inactivate the enzyme and build up the acetaldehyde concentrations to cause aversion to alcohol. Recently, DSF has gained prominence as a potent anticancer drug, owing to its pharmacological actions on multiple targets in tumor cells. It has been shown to trigger oxidative stress by generating reactive oxygen species (ROS) [2], abrogate the superoxide dismutase (SOD) activity [3] and activate the mitogen-activated protein kinase (MAPK) pathway [4]. DSF can reverse the resistance to chemotherapy drugs by inhibiting the P-glycoprotein (Pgp) multidrug efflux pump [5]. It was shown to inhibit the activation of nuclear factor-kB (NF-kB) a well-known resistance marker [4, 6]. In addition, DSF has been found to reduce angiogenesis because of its metal chelating properties, inactivation of Cu/Zn superoxide dismutase (SOD) and matrix metalloproteinases [3, 7]. DSF has also been shown to inhibit the proteasome [5], DNA topoisomerases [8], DNA methyltransferase (DNMT) [9] and glutathione S-transferase P1 (unpublished). More importantly, related to this study, our laboratory has demonstrated the ability of DSF and to inhibit O^6^-methylguanine DNA methyltransferase (MGMT), a DNA repair protein highly expressed in brain tumors [10]. MGMT normally functions to protect the genome against mutations generated by the pairing of O^6^-alkylguanines with thymine. Paradoxically, the higher MGMT content in gliomas serves to remove the mutagenic and cytotoxic O^6^-alkylguanines and confer tumor drug resistance and therapy failure [11]. Consistent with this observation, we have also found that different dithiocarbamates also retained the ability to inactivate MGMT [12]. Because DSF bears several properties, including the ability to cross the blood-brain barrier (BBB) [13], cytotoxicity against glioma stem cells [14], inhibition of various targets, including the MGMT and ALDH [15], there is significant interest in developing DSF for brain tumor treatment; in fact, a clinical trial has already been listed [16]. DSF with or without soluble copper (Cu-gluconate) is also undergoing clinical trials in liver cancer and other cancer types [17, 18].

After absorption, disulfiram is rapidly split into its half-molecule diethyldithiocarbamate (DDC), which readily complexes with copper in the stomach. Consequently, absorption and distribution via the gastrointestinal mucosa to the blood might involve both the parent drug and its copper diethyldithiocarbamic acid (DDC), which is unstable and is further degraded to form diethylamine and carbon disulfide [19]. DDC is also a substrate for phase II metabolism, which involves the formation of diethyldithiomethylcarbamate (Me-DDC) and the glucuronic acid of DDC. Me-DDC also undergoes oxidative biotransformation to diethylthiomethylcarbamate (Me-DTC) and is further oxidized to its corresponding sulfoxide and sulfone metabolites. Surprisingly, it was found that the plasma concentration of DSF after oral administration of a 500-mg tablet was below the limit of detection which explains the limitations of the DSF as a drug in a clinical setting [19–21]. Its extreme instability in the gastric environment and rapid degradation by the glutathione reductase in plasma appear to cause an unfavorable pharmacokinetics, which is hampering its further development as an anticancer drug [19].

The development of drug carriers, which deliver the drugs selectively to the site of action with a controlled rate of release has been a goal of drug therapy. Among various types of drug carriers, polymeric nanoparticles are advantageous in terms of stability, flexibility to control the rate of drug release and stealth behavior in circulation. Poly(Lactide-co-Glycolide) or PLGA is an FDA approved biodegradable polymer that is physically strong, highly biocompatible and with an established use for delivering drugs, proteins, and nucleic acids. A long clinical experience, favorable degradation characteristics and possibilities for sustained drug delivery are other advantages of PLGA. PLGA undergoes hydrolysis to yield the monomers (i.e., lactic and glycolic acid) which are endogenous and are metabolized by the body via Krebs cycle resulting in minimal systemic toxicity [22]. Polyethylene glycol (PEG) forms a hydrophilic coat over the hydrophobic PLGA core and protects the formulation from being engulfed by the reticuloendothelial cells (RECs) giving a stealth behavior and confers long circulation properties by acting as steric stabilizer [23]. PEG minimizes the ionic strength which stabilizes the nanoparticles from aggregation in the physiological solutions [23]. Polymeric nanoparticles with particle sizes within the range of 100 nm or less can take advantage of the enhanced permeability and retention (EPR) effect, which exists in areas of new blood vessel growth. For example, the EPR effect can be used to deliver anti-cancer drugs into tumor tissues *via* passive targeting [24]. EPR is also known to impart less toxicity because of the preferential accumulation of the passive formulations at tumor sites and limit off-target side effects [24–26]. Enhanced EPR effects prevalent in disrupted blood brain-barrier found in CNS cancers may further aid a greater entry of nanoparticles.

**Figure 1:**
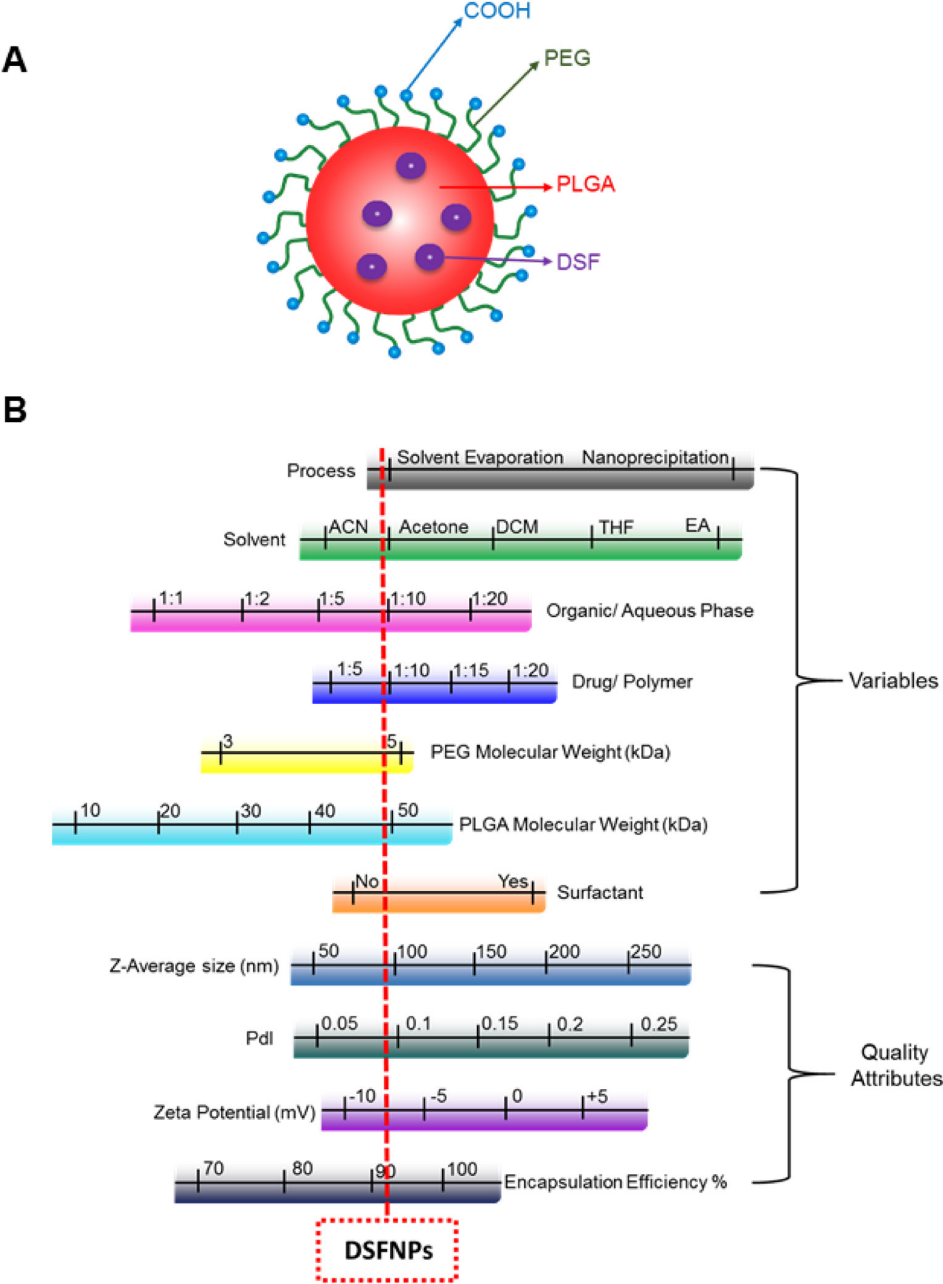
Representation of a disulfiram encapsulated nanoparticle and summary of optimization of DFSNPs (**A**) Schematic representation of a typical nanoparticle. (**B**). Ranges of formulation parameters and NP physicochemical properties evaluated during the development of DSFNPs. The red dotted line indicates the parameters of the finally developed DSFNPs, which was considered optimal for experimental and therapeutic purposes.

With the above considerations in mind, this study applied the most common and well-known mPEG: PLGA polymer for DSF encapsulation. PLGA forms a hydrophobic core in which the DSF gets encapsulated, and the surface of the core is covered with hydrophilic PEG chains (Figure 1A). There are a few reports of DSF nanoformulations with active targeting such as folate receptors targeted nanoparticles for breast cancer [25], lipid nanocapsules modified with cell penetrating peptide (TAT) for hepatic cancer [26], mPEG-PLGA/PCL nanoparticles for breast cancer [20], hot melt extruded and injection-molded PLGA millirods for GBM [27]. However, none of these have entered clinical trials; therefore, we considered a simpler approach to encapsulate DSF in mPEG: PLGA nanoparticles (DSFNPs). It is noteworthy that most of the FDA approved nanoformulations are made with PEG and all of them are passively targeted, despite the known advantages of specific delivery through active-targeting strategies [27, 28]. The current study prepared and optimized a formulation of DSF and characterized its physicochemical properties. The preferential accumulation of DSFNPs in the brain and the cellular pathways leading to the cytotoxicity were investigated. We also show DSFNPs exert strong antitumor effects in medulloblastoma orthotopic xenograft models.

## RESULTS AND DISCUSSION

### Preparation of DSFNPs and optimization of formulation

We used the single emulsion solvent evaporation technique, a proven and successful procedure for preparing DSF nanoparticles of hydrophobic drugs as described in Methods. The process and formulation variables play a key role in acquiring homogenous particles with a lower size and higher encapsulation efficiency. We found nanoparticles that were prepared using water-miscible solvents had better features compared to the water immiscible or partially miscible solvents. A ratio of 1:10 of organic/aqueous phase and drug/polymer resulted in optimal size and encapsulation. The presence or absence of surfactants with water-miscible solvents did not significantly influence on size or encapsulation. Among the various sizes of mPEG: PLGA polymers tested, the mPEG (5 kDa): PLGA (45 kDa) resulted in an optimal size and greater encapsulation efficiency. Figure 1B summarizes the formulation variables we tested in optimizing the physicochemical parameters of the DSFNPs. The procedural details and results of the DSFNPs optimization are provided in supplementary data (Supplementary Figures 1–3, Supplementary Table 1). Altogether, the formulation 1, prepared by solvent evaporation using acetone, mPEG (5 kDa): PLGA (45 kDa) polymer, without surfactant and at organic/aqueous phase and drug/polymer ratio of 1:10 gave the best size (∼70–80 nm), PDI (< 0.2) and %EE of >90%. We used this formulation1 for further characterization.

**Figure 2:**
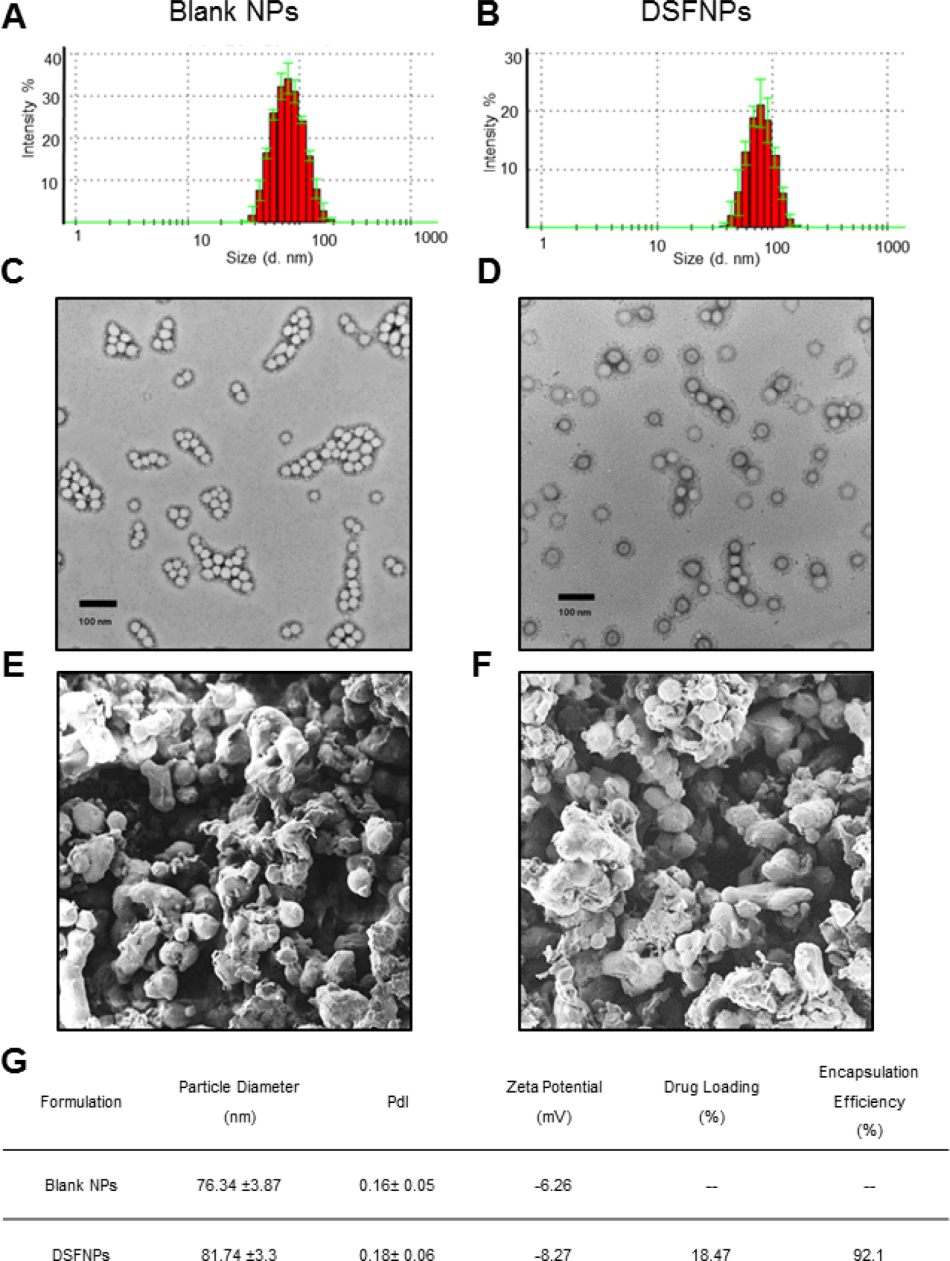
Characterization of mPEG-PLGA nanoparticles (**A, B**) The size, size distribution and surface morphology of the blank NPs and DSFNPs as determined by DLS (**C**, **D**) TEM and (**E**, **F**) SEM. (**G**) The size (d. nm), zeta potential, PDI and % EE of blank and DSFNPs. Data represent mean ± SD. Experiments were performed in triplicate (*n =* 3).

Both the blank and DSF encapsulated nanoparticles (DSFNPs) were analyzed for the size and the size distribution by dynamic light scattering (DLS) and representative size distribution plots are shown in Figure 2A, 2B. The monomodal distribution of the formulation was further confirmed with transition electron microscopy (TEM) (Figure 2C, 2D). Both DLS and TEM determined the average diameter of the blank NPs and DSFNPs in the range of 72–76 nm with a PDI of 0.16 and 0.18 respectively. The scanning electron microscopy (SEM) confirmed the spherical shape of the DSFNPs (Figure 2E, 2F). The zeta potentials of the blank NPs and DSFNPs were found to be −6.26 to −8.27 mV. The HITC- near-infrared dye incorporated nanoparticles (HITCNPs) were also found to have similar parameters as that of DSFNPs (Data not shown). The drug loading and encapsulation efficiency were determined to be 18.47% and 92.1% as summarized in Figure 2G.

### X-Ray diffraction analysis

**Figure 3:**
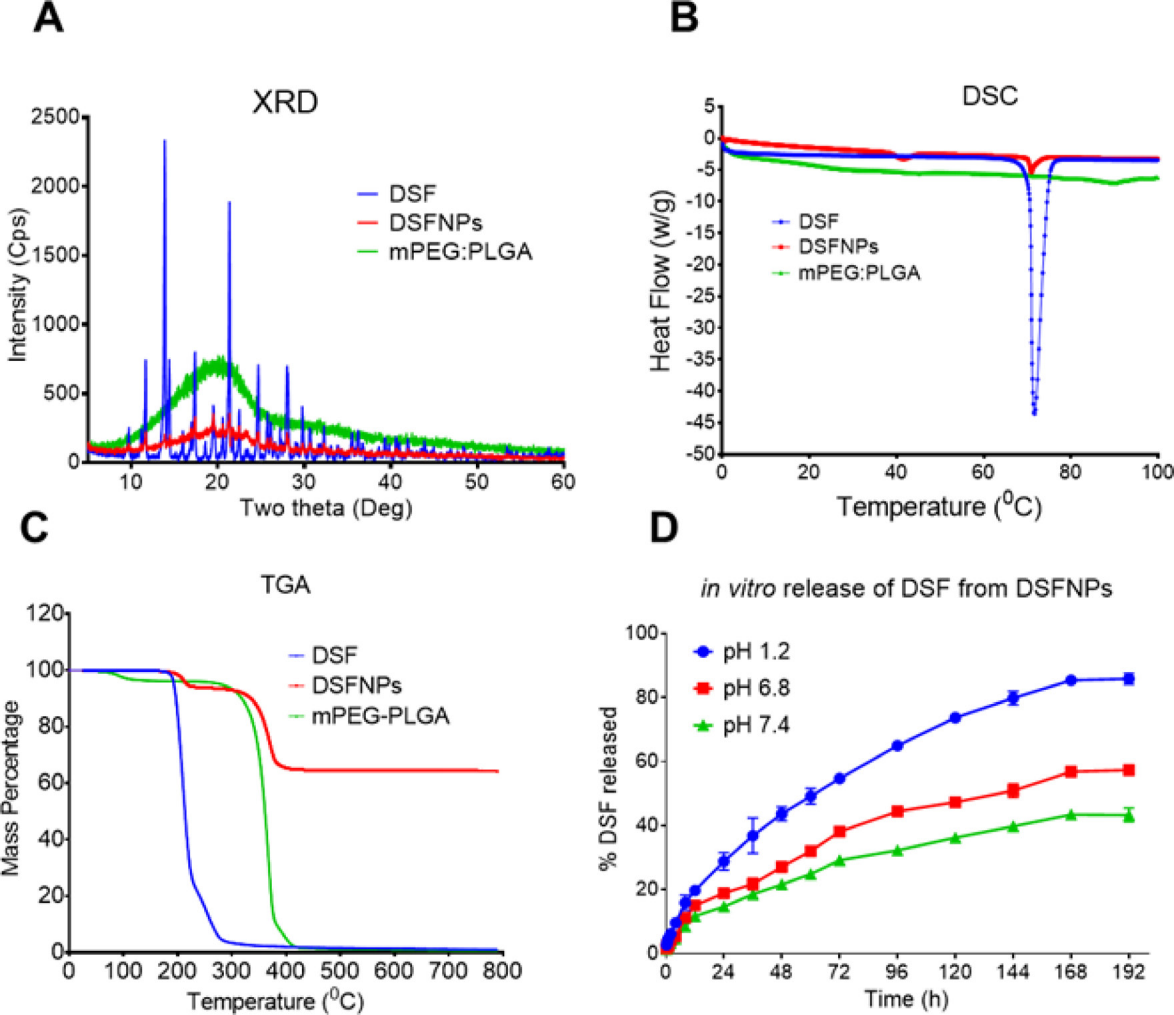
Physical characterization of DSFNPs Results from the (**A**) XRD; (**B**) DSC and (**C**) TGA of DSF, DSFNPs and mPEG: PLGA are represented. (**D**). The cumulative release kinetics of DSF from DSFNP in simulated gastric fluid (pH 1.2), simulated intestinal fluid (pH 6.8) and PBS (pH 7.4) are shown. The concentration units for DSFNPs and DSF used were equivalent in all experiments. Data represent mean ± SD. Experiments were performed in triplicate (*n =* 3).

XRD patterns of DSF, mPEG: PLGA polymer and DSFNPs are shown in Figure 3A. Characteristic diffraction peaks were observed for DSF free drug, whereas, no such intense diffraction peaks were apparent with the DSFNPs and the overall curve shape was similar to mPEG: PLGA. These results indicated complete encapsulation of DSF in the polymer matrix with the reduction in the crystallinity of the DSFNPs compared to the free drug.

### Differential scanning calorimetry

The thermograms define the physical states of the drug and the polymer and are useful in detecting any drug-polymer interactions within the polymeric network of the NPs. Differential scanning calorimetry (DSC) thermograms of DSF, mPEG: PLGA and DSFNPs are presented in Figure 3B. The DSF thermogram displayed an endothermic peak at 71.659°C and mPEG: PLGA did not show any endothermic peak in the temperature range tested, indicating the amorphous nature of the polymer. Whereas, DSFNPs showed a small endothermic peak at 70.911°C. These results indicated that there was no crystalline drug material on the surface of the formulation and confirmed its complete encapsulation. Further, it can be concluded that disulfiram in DSFNPs was present in the amorphous phase in a homogeneous dispersion within the PLGA matrix.

### Thermogravimetric analysis (TGA)

TGA analysis gave the degradation points of DSF, mPEG: PLGA and DSFNPs as shown in Figure 3C. The first depression in the curves was a measure of moisture content and we did not see any depression in DSFNPs until 300°C indicating no moisture in DSFNPs. DSF started losing the weight from 193°C (95%) and it reached 5% at 279°C. Polymer, mPEG: PLGA lost 5% of weight at 281°C and at 400°C it lost 95% of its weight. Whereas, the DSFNPs lost their weight by 5% at 217°C and 36% at 800°C leaving almost 64% of residue at the end.

### *In vitro* drug-release kinetics

*In vitro* drug release kinetics provide critical information about dosage form behavior and is a key parameter used to assess product safety and efficacy. The stability and controlled drug release profile of DSFNPs was assessed by measuring the cumulative release of DSF at physiological pH condition (pH 7.4), in simulated intestinal fluid (pH 6.8), and in simulated gastric fluid (pH 1.2). 14.5%, 19.4%, and 35.2% of the total DSF was released from the nanoparticles after 8 h at pH 7.4, 6.8, and 1.2 respectively, confirming that the majority of DSFNPs are stable under these conditions (Figure 3D). The cumulative drug release after 24 h was found to be 14.5%, 19.4%, and 35.2%, respectively, at above pH values and there was a steady release of DSF over the next nine days. These results provide strong evidence that DSFNPs are stable enough to allow a sustained release of the compound in both the stomach and intestine by protecting DSF from premature degradation. To gain a better understanding of the release profile of DSFNPs, we plotted the data as follows: cumulative % drug release vs time (Zero order kinetics model), log cumulative % drug remaining vs time (First-order kinetic model), cumulative % drug release vs square root of time (Higuchi model) and log cumulative % release vs log time (Korsmeyer- Peppas model). The R^2^ is a coefficient of determination, k is rate constant and *n* is release exponent. On the basis of best fit with the highest R^2^ value, it is concluded that in the optimized formulation of nanoparticles follow the Higuchi model (data is not shown), which has a large application in polymeric matrix systems [30]. The Korsmeyer- Peppas model revealed the release mechanism follows Fickian diffusion with release exponent value *n* ≅ 0.5 with all three buffers we tested.

### *In vitro* permeability and cellular uptake of DSFNPs

**Figure 4:**
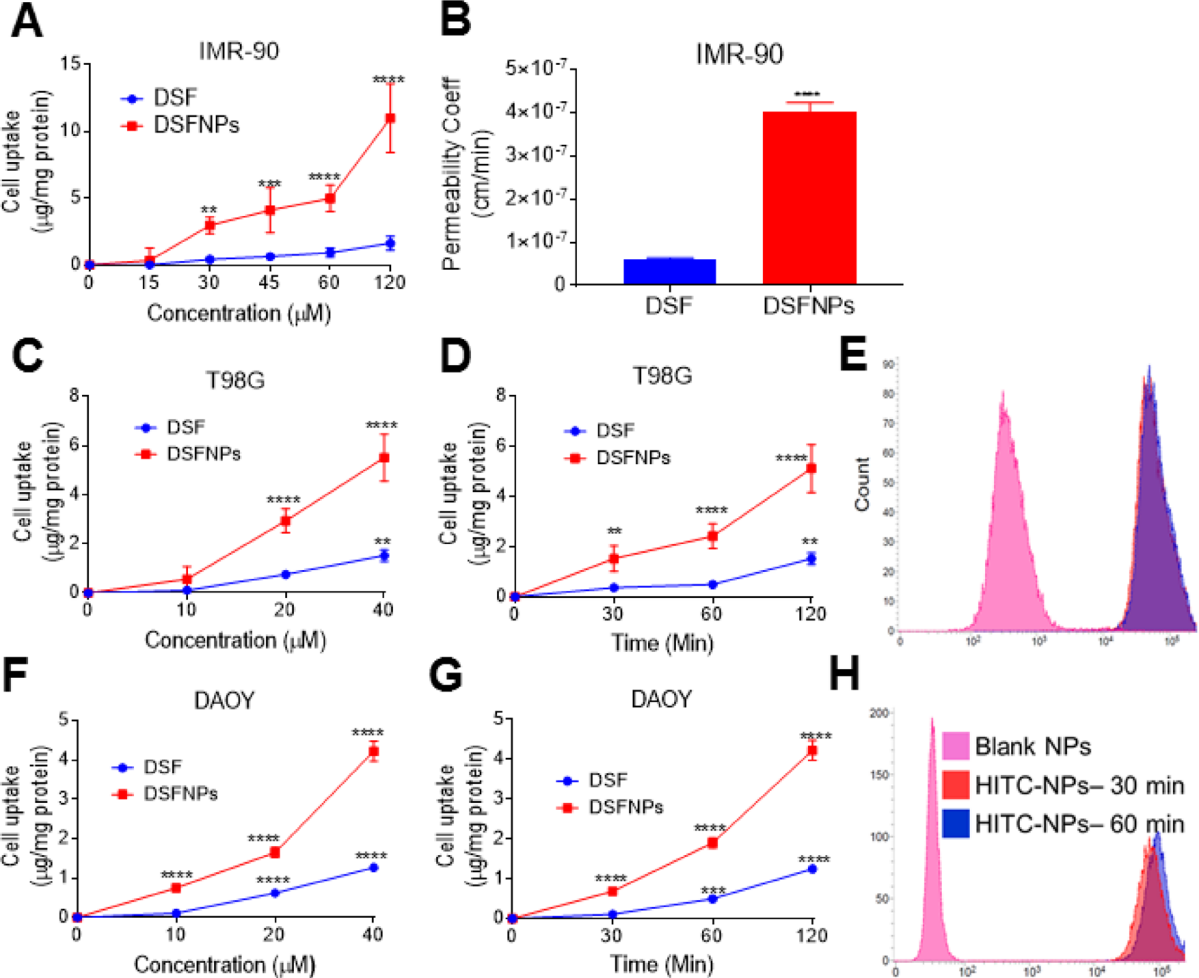
*In vitro* permeability and cellular uptake studies of DSFNPs (**A**) The permeability studies of DSFNPs through the IMR-90 stem cells characterized as an *in vitro* BBB model (**B**) permeability coefficient of DSF and DSFNPs in IMR-90 cells; (**C**, **D**) The cellular uptake of DSF and DSFNPs in T98G cells and (**F**, **G**) DAOY cells. T98G and DAOY Cells were treated with varying concentrations of DSF or DSFNPs for 2 h (**C, F**) or 25 μg/mL of DSF or DSFNPs for various times (**D**, **G**) DSF was extracted and quantified by HPLC and normalized to the protein content. (**E**, **H**) Incubation of cells with HITC-iodide dye encapsulated nanoparticles and a further measurement of fluorescence using FACS was performed. The FACS profiles and the peak shifts shown for both the T98G and DAOY cells confirmed the uptake of NPs. Data represent mean ± SD. Experiments were performed in triplicate (*n* = 3). ^*^, ^**^, ^***^, ^****^ indicate *p* < 0.05, <0.01, <0.001 and <0.0001, respectively one-way ANOVA followed by Dunnett multiple comparison test.

The effect of the encapsulation of DSF into mPEG-PLGA nanoparticles on the permeability of the drug was investigated using IMR-90 cells, a well-characterized *in vitro* model for BBB permeability studies [29, 31]. As shown in Figure 4A–4B, the transepithelial transport of DSF was significantly enhanced by the nano-delivery system, in a time and dose-dependent manner. There was a five-fold increase in DSF transport from the nanoparticle groups when compared to DSF alone in IMR-90 cells after 2 h incubation. The apparent permeability coefficients (*P*_app_) of DSF and DSFNPs were 6.037 × 10^–8^ cm/s and 4.01 × 10^–7^ cm/s respectively. The transepithelial electrical resistance (TEER) was not affected by DSF and DSFNPs throughout the experiment, suggesting that the increased DSF transport was not due to a decrease in the monolayer integrity or the opening of tight junctions.

### *In vitro* uptake of DSFNPs by cancer cells

Uptake of DSF and DSFNPs was investigated in the T98G and DAOY brain cancer cell lines. As shown in Figure 4D, 4G, the cellular uptake increased in a time-dependent manner for both DSF and DSFNPs. Compared to the free DSF, there was 2–3 -fold higher cellular uptake of DSFNPs in both cell lines after a 2 h incubation. We observed a similar trend in the experiments performed with different doses of DSF and DSFNPs (Figure 4C, 4F).

Further, the nanoparticle-induced enhancement of the cancer cell uptake of the encapsulated drug was confirmed using HITC encapsulated NPs (HITCNPs). The fluorescence of intracellular HITC was determined by flow cytometry. The intensity and shifting of the fluorescence peaks shown in Figures 4E, 4H indicate that there was a significant increase in the uptake of disulfiram after formulation when compared to HITC alone in both the cell lines.

### Real-time imaging of near-infrared (NIR) dye HITC (1,1′,3,3,3′,3′-hexamethylindotrycarbocyanine iodide)-encapsulated mPEG-PLGA nanoparticles in normal mice and tumor-bearing mice

**Figure 5:**
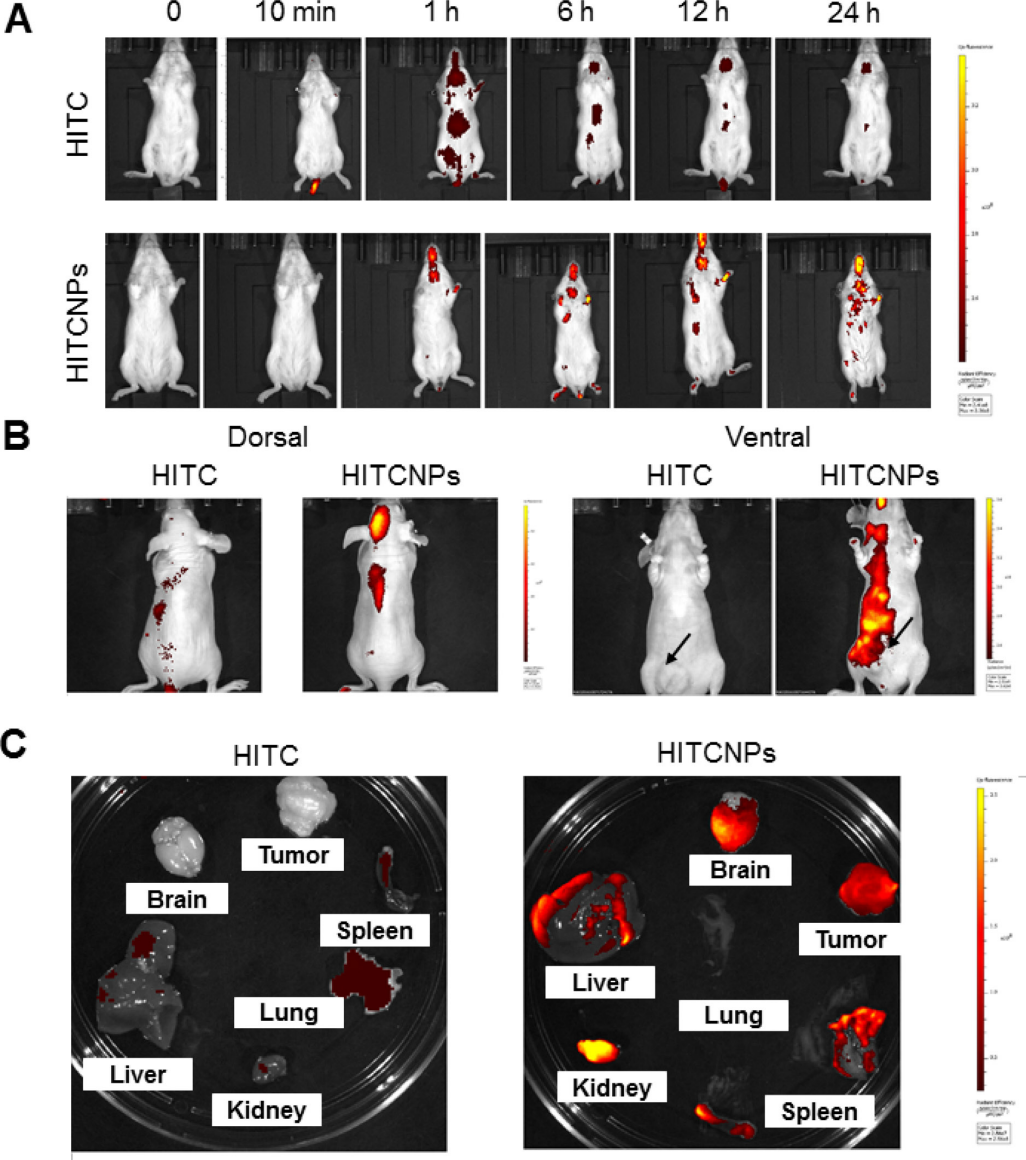
Real-time imaging of HITC labeled PEG-PLGA NPs (HITCNPs) *in mice* shows a preferential accumulation of particles in the brain (**A**) To study the fate of the NPs, normal mice (CD-1strain) were injected with the near infra-red (NIR) dye HITC alone or mPEG-PLGA nanoparticles labeled with HITC. The particle distribution as visualized by imaging the animals using a Calipers *In vivo* Imager. The representative photographs show that there was a time-dependent and preferential accumulation of NPs in the brain. Please note the yellow color represents the highest fluorescence intensity and the dye injected alone was eliminated over the time course. (**B**) The HITC-loaded NPs were also injected into a nude mouse bearing a subcutaneous HT29 colon tumor on the left flank. Again, a significant accumulation of the NPs in the tumor and the brain was evident. The yellow glow indicates the highest fluorescence localization. The subcutaneous tumor was generated on the ventral side of the animal and note the accumulation of HITCNPs therein. (**C**) *Ex vivo* fluorescence intensities of various organs from the nude mice injected with HITC –labeled NPs 24 h post-injection. The mice were euthanized, and the vital organs were extracted, washed twice with PBS, blotted dry and transferred to a petri dish. *Ex vivo* organ fluorescence intensities were recorded with an IVIS at similar imaging settings. The fluorescence associated with the liver, brain, tumor, lung, kidney, and spleen is well represented. All images are displayed on the same scale and the living imaging software was used to analyze the imaging data generated as described in Methods.

**Figure 6:**
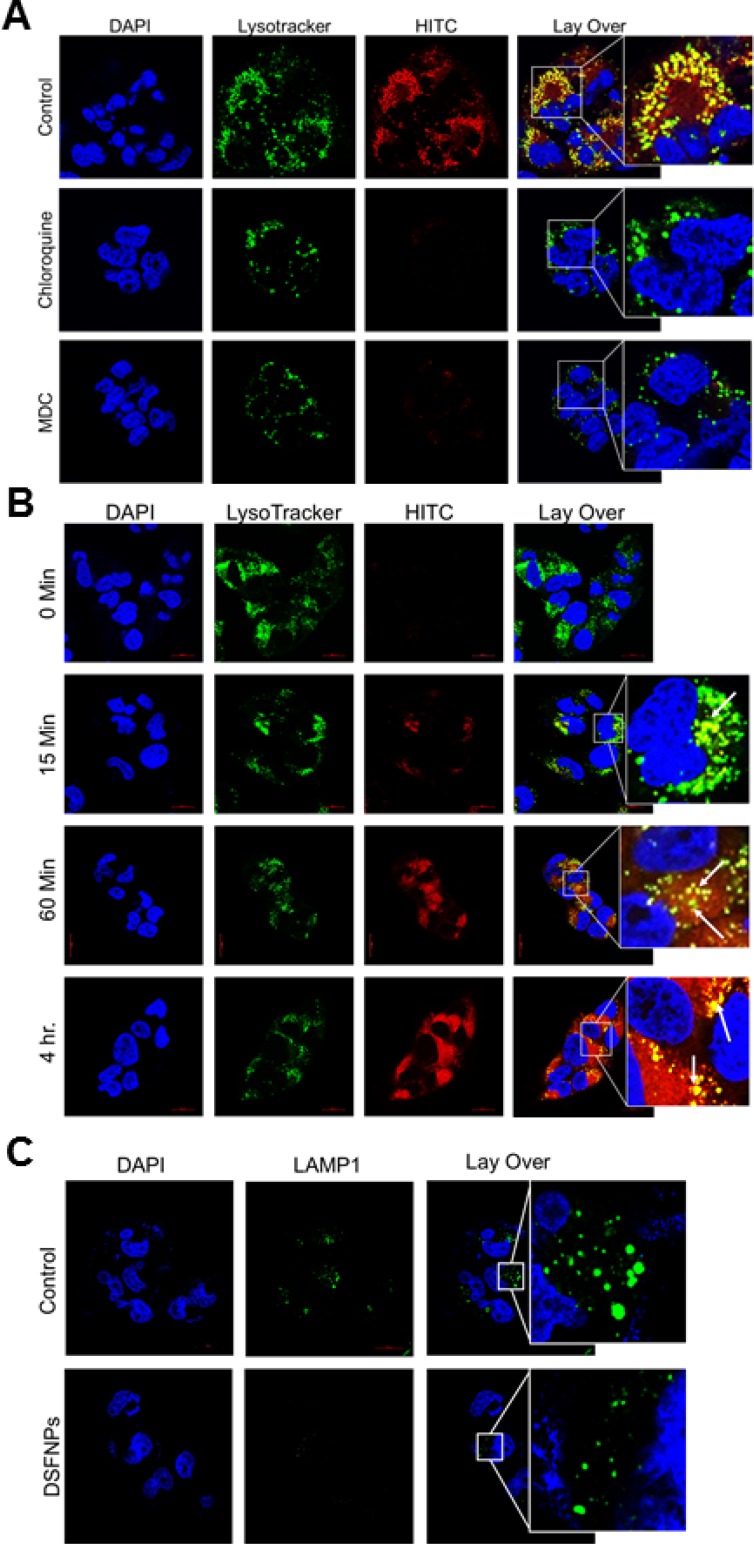
Clathrin-mediated endocytosis of HITCNPs and time-dependent accumulation of HITC-NPs in lysosomes in glioblastoma cells and evidence that loss of lysosomal membrane integrity mediates the drug release (**A**) The confocal photomicrographs show that following cellular internalization, the HITC-labeled NPs colocalize with the lysosomes in T98G cells. The first panel represents the cells treated with HITCNPs alone (red fluorescence) and next two represent cells pre-treated with chloroquine and MDC followed by incubation with HITCNPs. The cells were stained with the LysoTracker (green fluorescence), a marker for lysosomes. The overlaid confocal microscopy images show the colocalization of the lysosomal marker and the HITCNPs, represented by a yellow-orange punctate pattern. Note that both chloroquine and MDC strongly inhibited the internalization of DSFNPs. (**B**) Increased co-localization of HITC-NPs in lysosomes in T98G cells in a time-dependent manner. Lysotracker was used to label the lysosomes. A clear colocalization of the HITC-loaded NPs (red) with the lysosomes (green) is evident in the overlaid images showing a yellow-orange punctate pattern. (**C**). The decrease in the expression of lysosomal membrane protein LAMP1 in T98G cells treated with DSFNPs. Cells were stained with antibodies to LAMP1, a lysosomal membrane protein degraded by acid hydrolases. Decreased LAMP1 staining in DSFNP-treated cells is apparent.

To test our hypothesis that DSFNPs extravasate brain microvessels and deliver DSF across the BBB, the BBB-impermeable NIR fluorescent dye HITC was encapsulated into mPEG-PLGA nanoparticles and set up a noninvasive whole-animal fluorescence imaging method to trace the *in vivo* fate of the nano-formulation. The HITC dye is highly hydrophobic and was encapsulated similarly to DSF. Both the HITC dye and HITC dye encapsulated nanoparticles (HITCNPs) were administered intravenously to the normal mice (CD-1) through tail vein injections and allowed to circulate for up to 24 h. Figure 5 shows the real-time *in vivo* bio-distribution for HITC and HITCNPs in live animals. From the IVIS analysis, it is clear that HITCNPs penetrated the brain at 1 h and reached highest levels at 24 h. As, the free dye gets quickly removed from the body, and mice showed lower systemic fluorescence intensities compared to HITCNPs, and the difference was more pronounced at the 24 h time-point. Therefore, it is perceivable that a similar mechanism can be exploited for the entry of DSF to the brain at therapeutic doses. Next, we investigated the selective intratumoral accumulation of mPEG: PLGA nano formulation in tumor-bearing immunocompromised nude (*nu/nu)* mice. When the HT29 tumor volume was around 100 mm^3^, mice were injected intravenously with the HITC dye alone and HITCNPs through the tail vein. Similar to the CD-1 mice, a strong NIR fluorescent signal coming from the cingulate cortex of mice brain was observed at 24 h after intravenous injection with the HITCNPs (Figure 6B). Brains from control mice treated with HITC alone displayed no significant signal. Also evident is the accumulation of HITCNPs in the subcutaneous tumor formed on the abdominal ventral surface (Figure 6B right panel). To confirm the fluorescence signal we observed was truly coming from the brain and tumor, the mice were sacrificed 24 h- post injection and brain, tumor, and other major organs were dissected for *ex vivo* fluorescence imaging (Figure 6C). Organs with the brightest yellow color represent the highest HITCNPs accumulation. A strong fluorescence signal observed in excised brain and tumor of the HITCNPs treated mice confirmed that our nanoparticles formulation could cross the BBB, as well as selectively accumulate in the tumor microenvironment.

### DSFNPs enter the cells through clathrin-mediated endocytosis

Next, we attempted to study the mode of entry of our formulation into the cancer cells using the HITCNPs which mimic the DSFNPs. Receptor-mediated endocytosis (RME), also called clathrin-mediated endocytosis (CME) is one of the major means of transport of drugs or drug-loaded nanoparticles across the biological membranes. Further, CME reduces the non-discriminate uptake of toxic agents as well as enhances drug accumulation at the target site. NPs taken up by clathrin-dependent RME are typically destined for lysosomal degradation; whereas, clathrin-independent RME internalization leads to endosomal accumulation and sorting to a non-degradative path. After lysosomal degradation of the formulated polymer, drugs will be released into the cytosol through alterations of the lysosomal membrane integrity.

To confirm the clathrin-mediated endocytosis, we inhibited the CME by pretreatment of cells with well-established CME inhibitors chloroquine (CQ) or monodansyl cadaverine (MDC) followed by treatment with fluorescent dye loaded HITCNPs and stained with the LysoTracker. Blocking CME pathway resulted in reduced accumulation of HITCNPs as indicated by no colocalization of HITCNPs with lysosomal marker compared to CQ and MDC untreated cells (Figure 6A). Further, we observed a significant colocalization (represented with arrow marks) of HITCNPs with the lysosomes after 15 min incubation, and the fluorescence intensity was maintained as a function of time (15 min- 4 h) as shown in Figure 6B.

**Figure 7:**
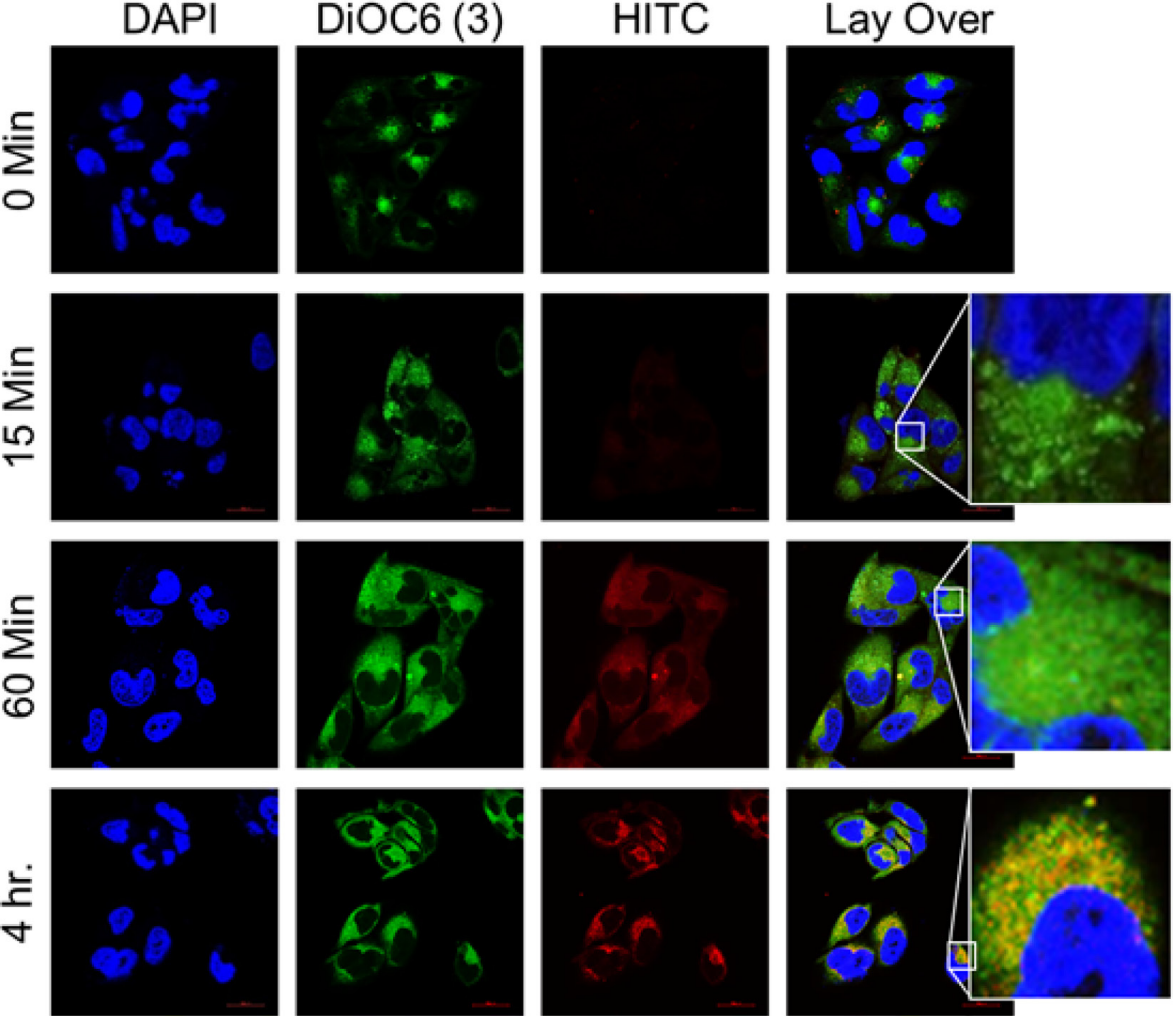
Co-localization of HITCNPs with mitochondria (**A**) Increased accumulation of HITCNPs in mitochondria in T98G cells in a time-dependent manner. Cells were treated with the DiOC6(3) and HITC-labeled nanoparticles as described in Methods. The overlaid images show the overlap of the mitochondrial membrane marker (DiOC6(3), green) with that of HITCNPs (red), as denoted by the yellow-orange punctate pattern, which is particularly strong at 4 h post-treatment.

Further, we studied the release of DSF from the formulation and its accumulation in mitochondria. The polymer usually gets degraded in the hostile lysosomal environment, releases the DSF, which in turn is discharged into cytosol through lysosomal rupture. Disruption of lysosomal integrity was examined by measuring immunofluorescence associated with LAMP1, a lysosomal membrane protein that protects lysosomes from the acidic hydrolases. After treatment with DSFNPs in T98G cells, a decreased LAMP1 immunofluorescence was observed when compared to the untreated cells indicating the loss of lysosomal membrane integrity (Figure 6C). Next, we found that the released HITC was selectively recruited to the mitochondria, which was confirmed by the co-localization of HITC fluorescence with DiOC6(3), a green fluorescent dye that selectively stains the mitochondria (Figure 7). The mitochondrial accumulation was highest at the 4 h time-point as indicated by a strong merging of fluorescence of HITC, and the mitochondrial tracker dye. Taken together these results confirm that mPEG-PLGA nano formulation undergoes CME, accumulates in lysosomes, upon polymer degradation, the drug will be released into the cytosol followed by its mitochondrial uptake.

### DSFNPs induce oxidative stress

Disulfiram, as a free drug, has been reported to induce ROS [32] and activate the MAPK pathway [2]. Therefore, we tested the ability of DSFNPs to induce ROS by staining the T98G and DAOY brain cancer cells with DCF-DA (2′,7′–dichlorofluorescein diacetate) or DHE (dihydroethidium). DCF-DA is a fluorogenic dye that measures the activity of peroxyl and hydroxyl radicals in the cells. The cell-permeable DCF-DA is deacetylated by cellular esterases to release a non-fluorescent intermediate, which upon interaction with ROS is oxidized to yield fluorescent DCF [32, 33]. DHE is a superoxide indicator, which exhibits blue- fluorescence in the cytosol until oxidized by superoxides, after oxidation it intercalates within the cellular DNA and stains the nucleus with a bright fluorescent red color with 500 and 580 nm excitation and emission wavelengths respectively.

**Figure 8:**
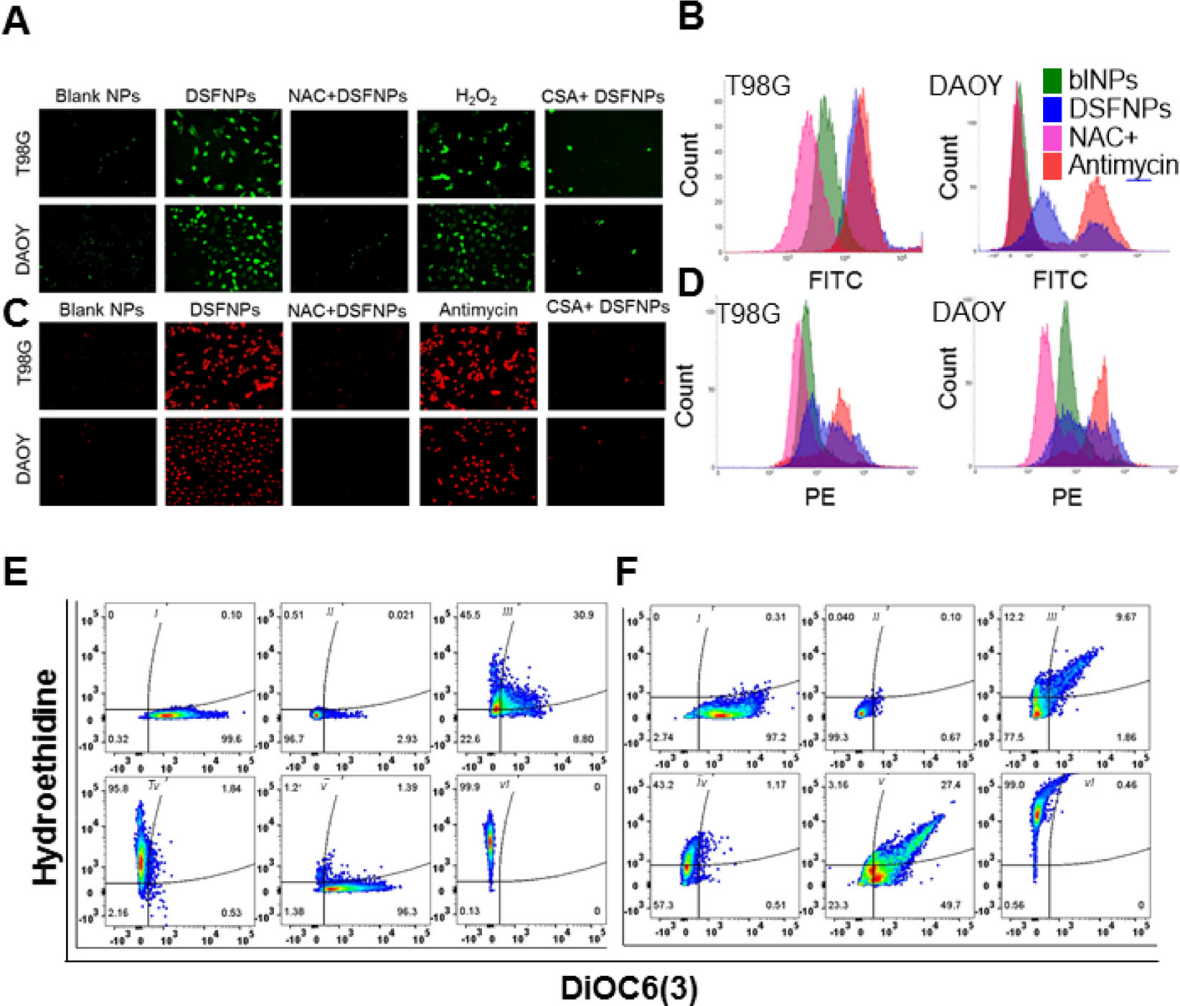
Evidence for the induction of ROS and loss of mitochondrial damage in DSFNPs treated brain tumor cells (**A-D**) Determination of ROS accumulation in T98G and DAOY cells treated with DSFNPs alone or pre-treated with N-acetylcysteine (NAC, 3 mM for 4 h) or cyclosporine A (CSA, 5 μM for 4h), and then exposed to DSFNPs. Cells were treated with DSFNPs for 24 h followed by staining with DCF-DA (**A**) or DHE (**B**) to show the induction of peroxides and superoxides respectively. H_2_O_2_ and antimycin, which are known to elevate ROS levels, served as positive controls. NAC, a free-radical scavenger, and cyclosporine-A, which promotes the integrity of mitochondrial membrane, were used as other controls and quenched the DSFNP-induced ROS. Cells were imaged using a fluorescence microscope. ROS-induction by DSFNPs was also confirmed by flow cytometry in T98G and DAOY cells with DCF-DA (**B**) and DHE (**D**). The peak profiles and shifts obtained with various treatments are shown in the histograms. The wavelength channels of FITC (fluorescein isothiocyanate) and PE (phycoerythrin) were used to analyze the DCF-DA and DHE-associated fluorescence. A significant increase in the fluorescence with DSFNPs was observed relative to the blank NPs, whereas, NAC pretreatment diminished the same. Antimycin alone treatment inducing ROS was a positive control. (**E, F**) Mitochondrial loss of permeability induced by DSFNPs. T98G (**E**) and DAOY (**F**) cells were costained with DiOC6(3) and DHE after treatment with DSFNPs to correlate the relationship between ROS-induction and loss of mitochondrial membrane integrity. DiOC6(3) is a lipophilic, cationic dye that stains mitochondria of live cells. (*i-* Control, *ii-* Control+ CCCP, *iii-* DSFNPs 12.5 μM, *iv*- DSFNPs 25 μM, *v-* NAC+ DSFNPs, *vi-* Antimycin). CCCP is a protonophore and uncoupler of oxidative phosphorylation is known to cause depolarization of the mitochondrial membrane. The flow cytometry profiles shown demonstrate a positive correlation between ROS induced by DSFNPs and loss of mitochondrial membrane polarity, which was further confirmed by NAC pretreatment. Antimycin A, a superoxide inducer was used as a positive control. Based on the cell populations, either ROS-positive (upper quadrants) and DiOC6(3)–negative mitochondrial membrane-impaired (left quadrants), it is clear that DSFNP-induced ROS disrupted the mitochondrial membrane integrity.

Microscopic examination of DCF-DA and DHE stained DSF or DSFNPs treated cells demonstrated a significant increase in the intensity of DCF and DHE staining as shown in Figure 8A, 8C respectively and Supplementary Figure 4A, 4C respectively. FACS analysis further confirmed a significant increase in ROS levels in drug-treated T98G and DAOY cells as shown in Figure 8B, 8D, and Supplementary Figure 4B, 4D. NAC pretreatment quenched and reversed these effects confirming the DSF mediated ROS elevation. H_2_O_2_ and antimycin were used as positive controls as peroxide and superoxide inducers respectively. These results demonstrate that DSF retains its oxidative stress-inducing ability after its formulation into DSFNPs. Further, DSFNPs exhibited superior oxidative stress induction when compared to the free DSF in both T98G and DAOY cells.

### DSFNPs induce loss of mitochondrial membrane integrity

It is well established that mitochondria play a major role in cell signal transduction through ROS production [32] and activating several stress-responsive pathways [33–35] leading to autophagy and apoptotic cell death [36, 37]. Loss of mitochondrial transmembrane potential affects the membrane integrity, and it releases ROS and triggers the initiation of the apoptotic cascade [37, 38]. Consequently, the apoptotic inducers such as AIF (apoptosis-inducing factor) and cytochrome C leak out through the damaged mitochondrial membrane and translocate into the nucleus to trigger the apoptotic chain of events [39, 40]. To establish the effect of DSF-induced ROS on mitochondria, we pre-treated T98G and DAOY cells with cyclosporine-A. After 4 h, they were exposed DSFNPs for one day and then stained with DCF-DA or DHE (dihydroethidium). Cyclosporine A is an established inhibitor of mitochondrial permeability transition [40] (MPT) pore by binding with cyclophilin D [41, 42] and thereby upholds the integrity of the mitochondrial membrane. We observed a strong decrease in the fluorescence intensity of DCF-DA and DHE in cyclosporine-A pretreated cells suggesting that ROS induced by DSF were significantly diminished, whereas, in the absence of cyclosporine A, the cells showed intense fluorescence with both free-radical scavengers. These observations encouraged us to probe if the mitochondrial membrane was depolarized by DSFNPs to release ROS. To confirm this, we adopted a flow cytometric method for the quantitative measurement of mitochondrial transmembrane potential (ΔΨ_m_) using 3,3′-Dihexyloxacarbocyanine Iodide [DiOC_6_(3)]. We used carbonyl cyanide m-chlorophenylhydrazone (CCCP), a potent mitochondrial oxidative phosphorylation uncoupler, which renders the mitochondrial inner membrane permeable to protons as a positive control for membrane depolarization. We co-stained the cells with DHE to correlate DSF-induced ROS and decrease in the mitochondrial membrane potential (ΔΨ_m_). In this study, we have observed a significant decrease in mitochondrial trans-membrane potential in DSFNPs treated cells (T98G and DAOY cells) when compared to the untreated cells (Figure 8E, 8F). These results were further validated by employing negative and positive controls of oxidative stress, N-acetylcysteine (NAC) and antimycin A respectively. NAC pretreatment diminished the effect of DSFNPs on mitochondrial membrane depolarization, and antimycin potentiated the DSFNPs effect. Taken together, these data clearly demonstrate the interlink between DSF induced ROS and loss of mitochondrial membrane potential, which leads to increased leakiness of mitochondria.

### ROS induced by DSFNPs activate the MAPK pathway

**Figure 9:**
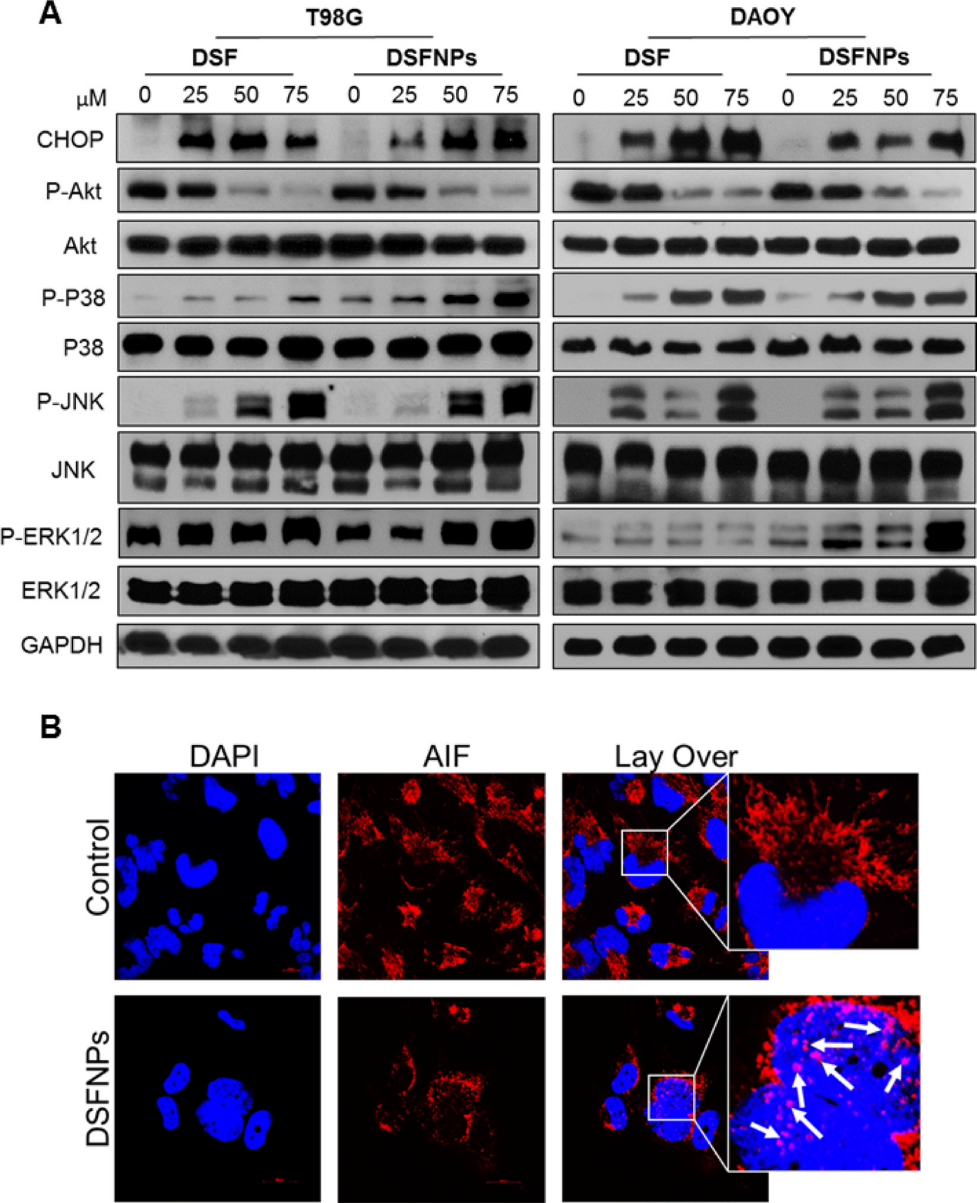
DSFNPs induce MAP-Kinase pathway components and nuclear translocation of AIF (apoptosis-inducing factor) (**A**) Cells were treated with DSF or DSFNPs at different concentrations for 24 h and western blotting using specific antibodies was performed. Both the DSF and DSFNPs increased the expression of CHOP and phosphorylation of ERK, p38, and JNK and blocked the phosphorylation of AKT in a concentration-dependent manner. GAPDH was used as loading control. (**B**) Nuclear translocation of AIF in T98G cells induced by DSFNP treatment for 24 h. Cells were analyzed by confocal microscopy after immunostaining with antibodies to AIF. Arrows indicate the translocation AIF into the nucleus. AIF is a mitochondrial protein and migrates into the nucleus to initiate the caspase-independent apoptosis by causing DNA fragmentation and chromatin condensation.

Mitogen-activated protein kinase (MAPK) signaling pathway is well known to play a critical role in diverse cellular processes including the regulation of endoplasmic reticular stress, cell proliferation, differentiation, autophagy [43] and apoptosis [2]. Further, it is well-known that reactive oxygen species (ROS) are potent activators of the c-Jun N-terminal kinase (JNK) through oxidative inactivation of endogenous JNK inhibitors, such as JNK phosphatases and GST-pi [42–44]. Figure 9A represents the effect of DSF and DSFNPs on the activation of the MAPK pathway. Though the basal protein expression of JNK, P38, and ERK1/2 was not affected much by the treatment, the expression of phosphorylated JNK, P38 and ERK1/2 increased persistently and reproducibly. We observed a corresponding decrease in phospho-AKT levels. However, the AKT protein levels remained constant. We also observed a significant increase in mitochondrial stress-sensitive protein CHOP.

The relationship between MAPK pathway and AKT is intriguing. Even the role of AKT in apoptosis is confusing. Activation (phosphorylation) of AKT renders tumor cells resistant to several chemotherapeutic drugs [45, 46]. It is reported that AKT activation occurs as a transient response of the cells undergoing apoptosis as a self-defense mechanism, being a pro-survival marker. However, an eventual decrease in AKT phosphorylation may prove necessary, for the overall induction of apoptosis [47].

### DSF induces nuclear translocation of apoptosis-inducing factor (AIF)

The above observations clearly suggest a role for the operation of apoptotic pathways in mediating the DSF cytotoxicity. It was reported that DSF inhibits caspase family proteins [46], on the other hand, we observed upregulation of proapoptotic proteins and downregulation of antiapoptotic proteins (Figure 9B), which strengthened the observation of Carmody *et al.* [47] and prompted us to check expression and localization of caspase-independent mitochondrial apoptotic protein such as the AIF [48–50]. Immunofluorescence microscopy revealed the subcellular co-localization of AIF with the nucleus in the T98G cells treated with DSFNPs for 24 h compared with the controls.

### DSFNPs induce apoptosis

**Figure 10:**
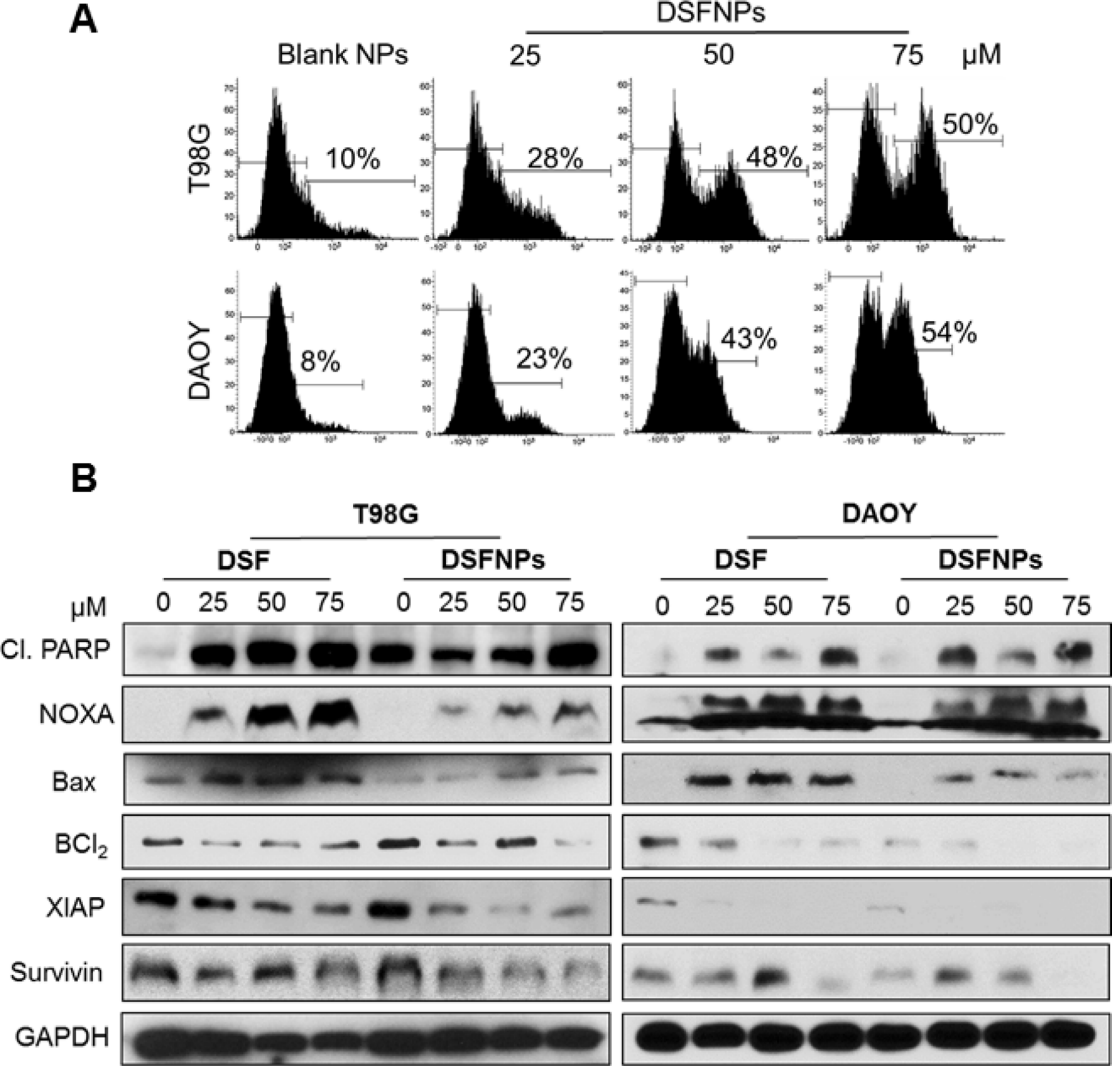
DSFNPs trigger apoptosis in brain tumor cells (**A**) Flow cytometric histograms of T98G and DAOY cells stained with FITC-annexin V after treatment with DSFNPs are shown. The shift in the peak to the right indicates the increase in the apoptotic cells in a concentration-dependent manner. The % of annexin-positive cells in the early-apoptotic phase are indicated. Annexin V is a member of the annexin-family of proteins that binds to phosphatidylserine in early-apoptotic cells. FITC-conjugated annexin V was used to detect the apoptosis using flow cytometry. Increase in the fluorescence indicates the increased number of the apoptotic cell population. (**B**) Expression of apoptotic and anti-apoptotic regulatory proteins after DSF or DSFNP treatment. Cells were treated with DSF and DSFNPs at different concentrations followed by western blotting. Both the free and encapsulated drug increased the expression of apoptotic proteins (cleaved PARP, Bax, and NOXA) and decreased the expression of anti-apoptotic proteins (BCl_2_, XIAP, and Survivin) in a concentration-dependent manner. GAPDH was used as loading control.

To determine the mechanism of cytotoxic effects observed with DSFNPs, Annexin-V/FITC apoptosis assay was performed by a flow cytometer in T98G and DAOY cancer cells. We observed a substantial increase in annexin-V positive cells with both cell lines compared to the untreated controls in concentration-dependent manner (Figure 10A).

Next, the changes in the protein expression of various pro- and anti-apoptotic proteins were quantitated by western blot analysis. DSFNPs treatment resulted in a significant increase in the expression of pro-apoptotic markers such as cleaved-PARP, NOXA, Bax proteins and corresponding decreases in the levels of pro-apoptotic proteins. Nevertheless, in agreement with the report by Nobel *et al.*, we observed a decrease in caspase expression [46] raising the possibility of caspase-independent apoptosis mediated through mitochondria by DSFNPs (Figure 10B) [50].

### *In vitro* cytotoxicity of DSFNPs

**Figure 11:**
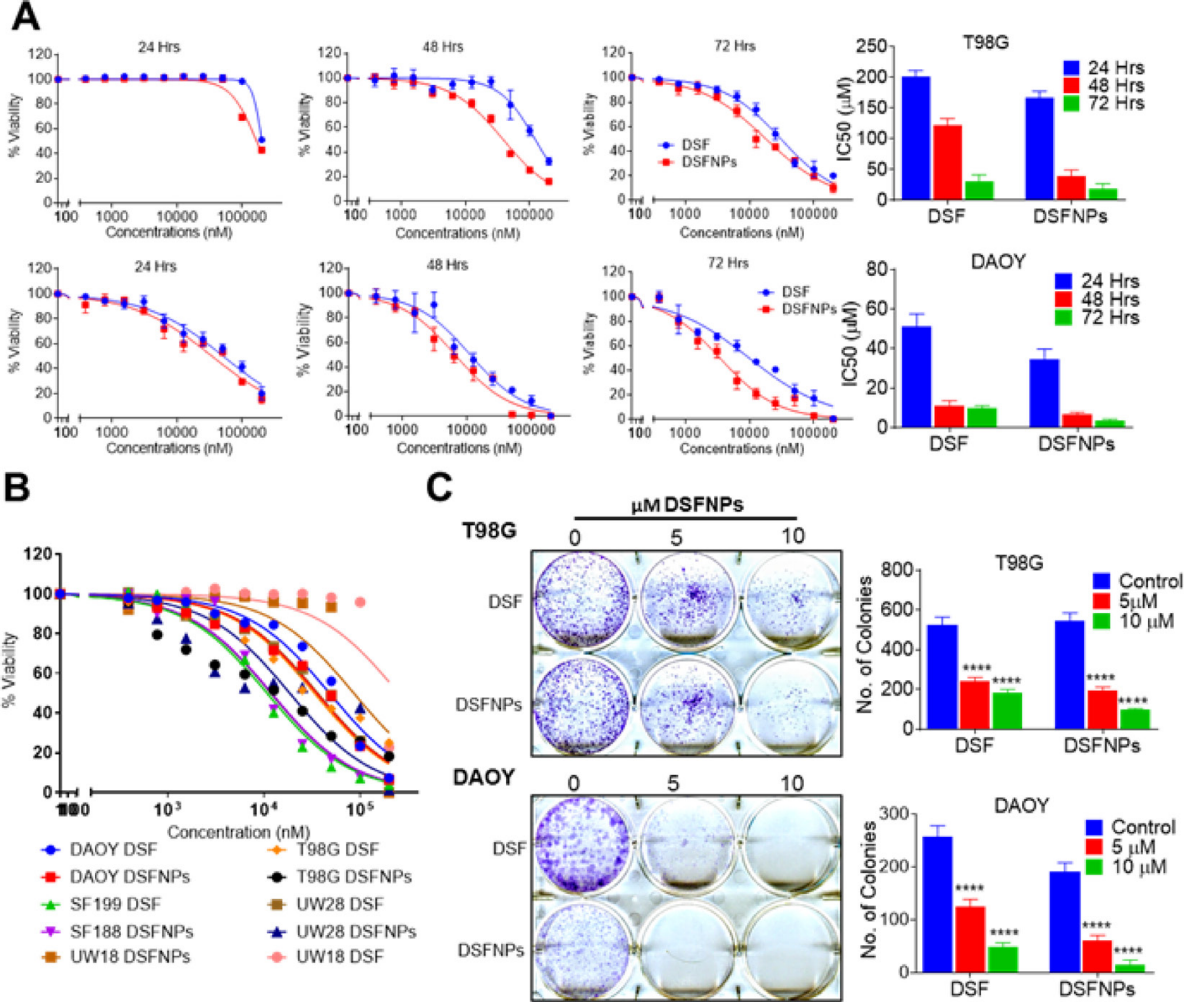
DSFNPs induce cytotoxicity in brain tumor cells to the same extent or more efficiently as DSF (**A**) Effect of DSF and DSFNPs at varying concentrations and time in T98G and DAOY cells. (**B**) Cytotoxicity of DSF and DSFNPs against other brain tumor cell lines (SF188, UW28, UW18) at 72 h. A fluorescence assay using resazurin was used. (**C**) The suppressive effect of DSF and DSFNPs on colony formation of T98G and DAOY cells. Representative images of the colony-forming assay are shown on the left panel. Colonies were counted using ImageJ software and plotted as bar graphs in the right panel. Data represent mean ± SD. Experiments were performed in triplicate (*n =* 3). ^****^ indicate *p* < 0.05, <0.01, <0.001 and <0.0001, respectively one-way ANOVA followed by Tukey’s multiple comparison test.

In continuation, the *in vitro* cytotoxicity of DSF and DSFNPs in various brain cancer cells were compared. Blank nanoparticles did not show any cytotoxicity among the cell lines tested (data not shown). The cytotoxic effects of DSF and DSFNPs in a time-dependent manner with 24 h, 48 h, and 72 h time points were determined, and the results are shown in Figure 11A. The IC_50_ values of free DSF in T98G cells were 201, 122, and 30 μM and in DAOY cells were 51, 11 and 9 μM at 24, 48, and 72 h, respectively. The IC_50_ values of DSFNPs in T98G cells were 167, 39 and 18 μM and in DAOY cells were 34, 6 and 3 μM at 24, 48 and 72 h, respectively. The IC_50_ values of DSF were slightly higher compared to DSFNPs, and the difference in IC_50_ between DSF and DSFNPs increased over the time from 24 to 72 h indicating the sustained release of DSF. We have already shown that DSF, as a free drug can quickly inactivate the MGMT DNA repair in xenografted brain tumors [10]; it should be noted that DSFNPs function as a drug reservoir and a long-term supply is expected to augment the antitumor efficacy. The cytotoxic profiles of DSFNPs against other brain cancer cells were as represented in Figure 11B.

In agreement with the extent of cytotoxicity noted above, the clonogenic survival assays in both T98G and DAOY cells revealed a similar trend of cell killing by DSFNPs, at an IC_20_ concentration. The decrease in the number of colonies was statistically significant with increased concentrations of free and encapsulated DSF, though, the differences between them were marginal (Figure 11C).

### Demonstration of *in vivo* efficacy of DSFNPs in an intracranial medulloblastoma xenograft model

**Figure 12:**
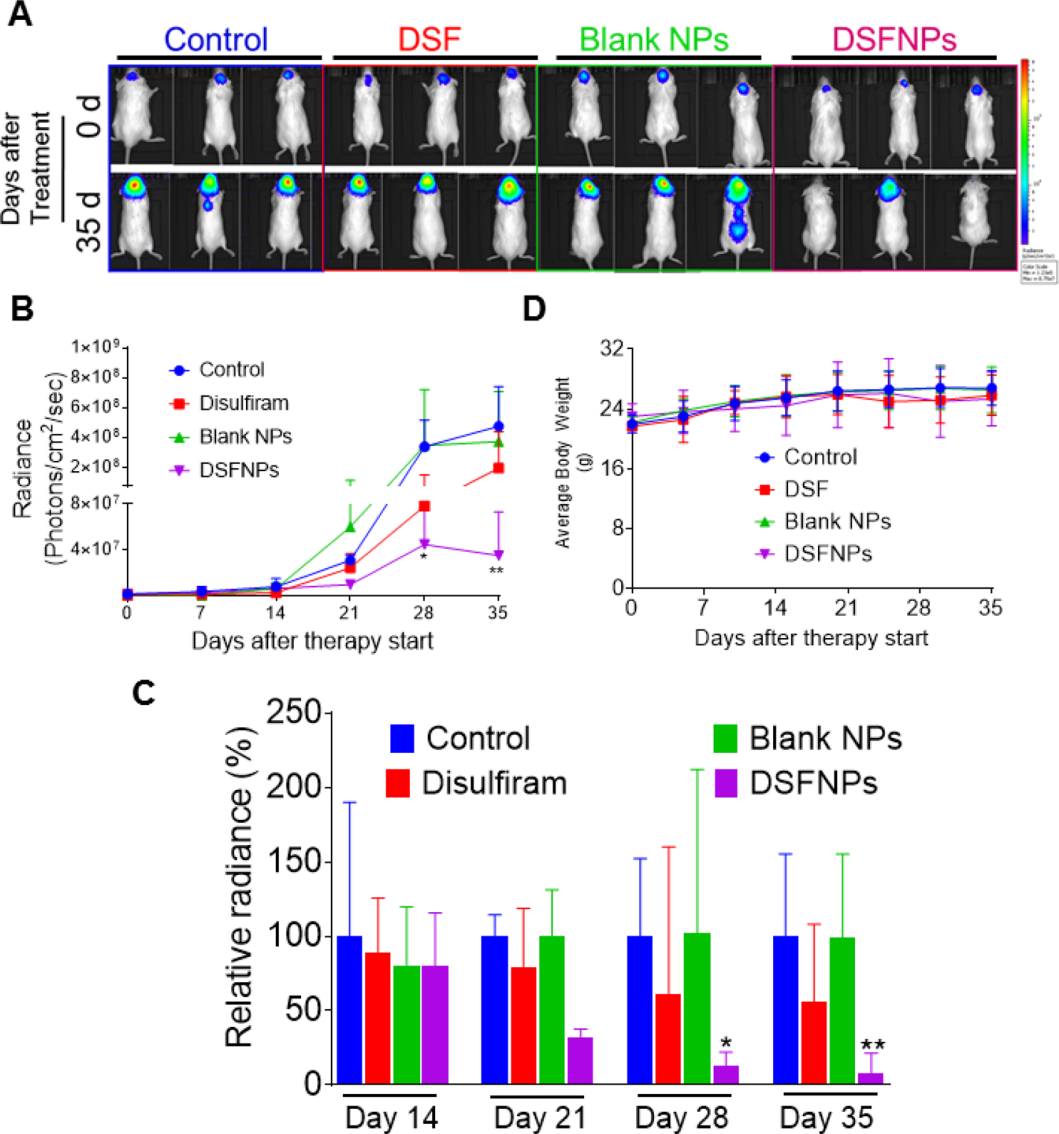
Strong antitumor efficacy of DSFNPs relative to DSF in DAOY human medulloblastoma intracranial xenografts (**A**) Representative images of bioluminescence acquisition of brain tumor at the beginning and end of therapy. Luciferase-expressing DAOY cells were injected into the brains of immunocompromised NCG mice as described in Methods. DSF or DSFNPs at 30 mg/kg, nanoparticles without DSF loading or the vehicle alone were given to the mice *i.p.* three times a week, and IVIS imaging was performed once every week. The bioluminescence was compared on a common scale ranging from 1.23 × 10^5^ to 8.75 × 10^7^ as shown in the color intensity bar (blue to red) on the right. The results showed a significant decrease in tumor burden in mice treated with DSFNPs, but not with DSF. (**B**) Average time-dependent changes in bioluminescence radiance in DAOY xenografts receiving therapy over 5 weeks. The radiance was processed and computed using the LiveImage software. Data represent mean ± SD, *n =* 6. ^*^ indicates significance at *p* < 0.05 and ^**^p < 0.01; one-way ANOVA followed by Dunnett’s multiple comparison tests were performed. (**C**) Changes in relative radiance in the treatment groups over days 14 to 35 are shown in the bar graphs. The average radiance of the control group mice was considered 100% at each time point. The data are expressed as mean ± SD. (**D**) The average body weights of the mice were plotted against the treatment period.

To evaluate and compare the anticancer efficacy of DSFNPs versus the free DSF in an orthotopic brain cancer xenograft setting, we first used lentiviral transfection of luciferase and developed a DAOY human medulloblastoma cell line with a high level of stable luciferase expression. These cells were implanted by stereotaxic injection into mice brains to generate intracranial xenografts and the tumor growth was assessed by quantitative bioluminescence using an *in vivo* imaging system. The tumor-bearing animals were divided into four groups and received just the vehicle, blank nanoparticles, DSF or DSFNPs. DSF (30 mg/kg) or equivalent amount of DSFNPs were administered i.p. three times a week for 5 weeks and the results are shown in Figure 12. The representative color luminescent images representing the spatial distribution of photon counts in the brain shows that disulfiram alone or blank nanoparticles did not affect the luciferase expression on day 35 of treatment, however, the DSFNPs, on the average showed a marked loss of bioluminescence (Figure 12A). The quantitative changes in average bioluminescence radiance calculated from 6 animals over the therapy period are drawn in Figure 12B; DSF, in its unencapsulated form, induced only a marginal, but discernible effect on tumor growth on days 28 and 35. In contrast, the DSFNPs elicited a strong and significant tumor growth inhibition, with a maximal 7-fold more tumor cell killing than the free drug during the same period. The relative % decrease in bioluminescence units shown in Figure 12C provides a better representation of the antitumor effects observed in our study. Compared to DSF, DSFNPs were 2.5, 4.6 and 7.5 times more potent in inhibiting the tumor growth on days 21, 28 and 35 respectively. These kinetics also suggest that DSF or DSFNPs as a single agent are likely to exert a delayed antitumor effect and extended cycles of treatment may be needed. There were no significant changes in the average body weight of the mice at the given dose in any group, suggesting that the treatment did not exert undue adverse effects to the host organs (Figure 12C). Collectively, the xenograft study justifies our objective of encapsulating the DSF in nanoparticles for brain tumor therapy.

## CONCLUSIONS

**Figure 13:**
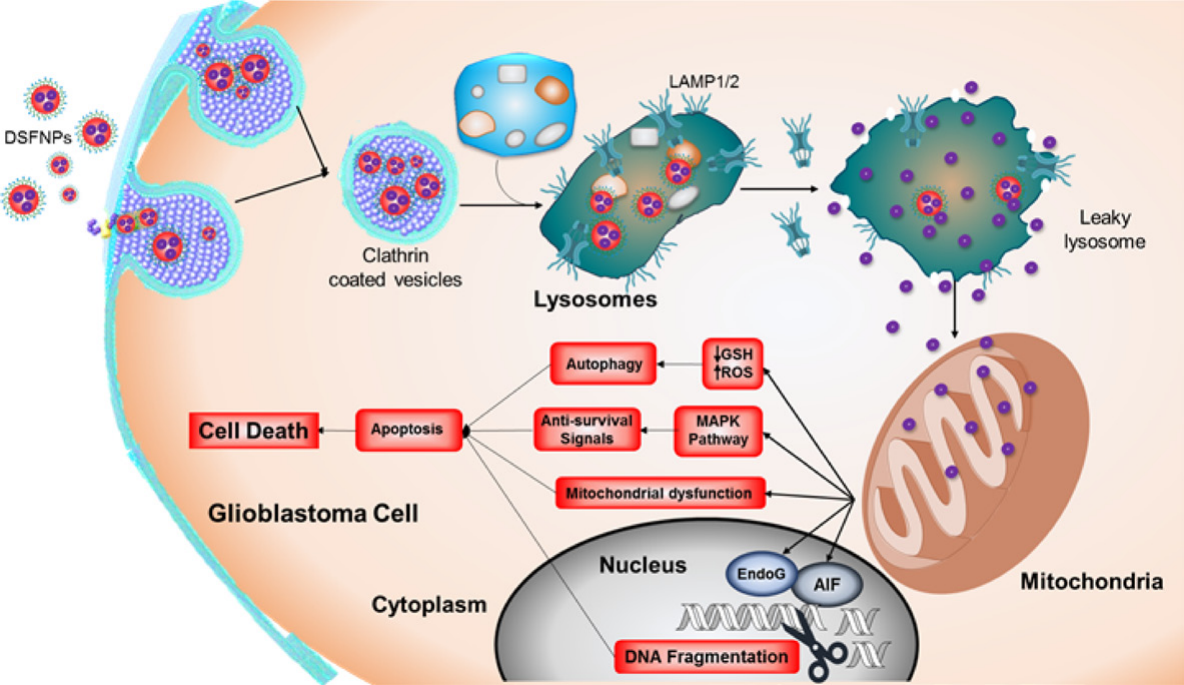
Schematic diagram showing the fate of DSFNPs and sequence of events leading to cytotoxicity as revealed by this study Uptake of the DSFNPs by receptor-mediated endocytosis, their accumulation in lysosomes, lysosomal breakdown, drug entry into mitochondria, the redox events culminating in the killing of brain tumor cells are represented.

Current therapies for malignant brain tumors are not effective, and an urgent need exists for designing improved treatment strategies. In this study, a nanoformulation containing DSF was successfully prepared using mPEG-PLGA as polymer, and the formulation was optimized to achieve therapeutically-relevant parameters, including a lower particle size, good polydispersity index and higher entrapment efficiency. The DSFNPs were further characterized for the surface, physical and thermal properties along with stability, permeability and drug uptake using appropriate techniques. A near-infrared dye encapsulated formulation with similar characteristics as that of DSFNPs revealed that the formulation selectively accumulates in the brain and in subcutaneous xenografted tumors. These results suggested that the formulation was able to efficiently deliver DSF to the brain with an increased plasma half-life. While the smaller size of the particles may facilitate the transport across the BBB, an enhanced permeability retention in the tumor vasculature [24, 54] may aid a preferential accumulation of these formulations in cancer tissues. A hypervascularity, incomplete vascular architecture, secretion of vascular permeability factors leading to extravasation within the malignant tissues and absence of lymphatic drainage for clearance may all contribute a greater accumulation of DSFNPs in brain cancers. The DSFNPs were able to induce killing of brain tumor cells and elicit various molecular alterations and apoptosis in a potent manner, with a marginally superior efficacy compared to the un-encapsulated free drug. However, it should be noted that disulfiram was released in a sustained fashion over a long time from the nanoparticles and in essence, the cytotoxic events observed were achieved at lower drug concentrations. In fact, the vastly superior antitumor effects of DSFNPs (compared to DSF by itself) we observed in orthotopic xenograft studies are consistent with this notion and justify the use of the nano-formulated drug in a clinical setting. Further, we were interested to see the downstream effects of DSFNPs after their entry into cells. A schematic representation of the uptake and cytotoxic mechanisms of DSFNPs in glioblastoma cells, as revealed by the current study is shown in Figure 13. We found the DSF nanoparticles to internalize through clathrin-coated vesicles, and accumulated in lysosomes and subsequently in mitochondria. Mechanistically, DSFNPs through, a release of DSF in a sustained manner induced ROS and loss of mitochondrial transmembrane potential. DSFNPs also activated the MAPK pathway and induced nuclear translocation of AIF to trigger apoptosis. DSFNPs induced cytotoxicity and exerted potent anti-clonogenic effects against T98G and DAOY cells (Figure 11). Based on these findings, studies are underway to determine the antitumor effects of DSFNPs with or without copper gluconate and temozolomide in glioblastoma orthotopic xenografts.

## MATERIALS AND METHODS

### Cell lines and cell culture

Human brain cancer cell lines DAOY and T98G were obtained from the American Type Culture Collection (ATCC, Rockville, MD). T98G and DAOY cells were cultured in Dulbecco’s minimum essential medium (DMEM) (Corning) supplemented with 10% fetal bovine serum (Atlanta Biologicals Inc.) and antibiotics. All cells were cultured in a humidified atmosphere with 5% CO_2_ at 37^°^C.

### Animals

Female CD-1 mice, female athymic nude (nu/nu) mice, and the triple immune-deficient NCG (NOD CRISPR Prkdc Il2r gamma) mice were obtained from Charles River Laboratories. All animal procedures were performed according to the Institutional Animal Care and Use Committee (IACUC) guidelines.

### Chemicals, antibodies, and other reagents

Disulfiram was purchased from Sigma (St Louis, MO). The m-polyethyleneglycol-polylactic-glycolic acid (mPEG-PLGA) polymers were purchased from Advanced Polymer Materials Inc. (Montreal, Canada). Solvents were purchased form Fisher Scientific. The near-infrared dye, 1,1′,3,3,3′,3′- Hexamethyl indotricarbocyanine iodide (HITC), 2′,7′-dichlorofluorescein diacetate (DCF-DA), chloroquine, cyclosporine A (CSA) and rest of the chemical reagents were purchased from Sigma (St Louis, MO). All antibodies were purchased from Cell Signaling Technology (Danvers, MA). Dihydroethidium (DHE), 4,5-dihydroxy-1,3- benzenedisulfonic acid disodium salt monohydrate (tiron), N-acetyl cysteine (NAC), 3,3′- Di-n-hexyloxacarbocyanine iodide (DiOC6(3)) and carbonyl cyanide 3-chlorophenyl hydrazine (CCCP) were from Thermo Fisher. The concentrations of the above reagents used for the study were 5 μM DHE, 25 μM DCF-DA, 1 mM tiron, 3 mM NAC, 2 nM DiOC6(3), 50 μM CCCP and 5 μM cyclosporine A. Lysotracker, Alexa 488- or 594-conjugated secondary antibodies were from Molecular Probes (Carlsbad, CA).

### Preparation of DSF loaded biodegradable PEG-PLGA nanoparticles (DSFNPs)

Single emulsification-solvent evaporation was used to encapsulate DSF into mPEG: PLGA matrix. Nanoparticles formulated in this way contain hydrophobic PLGA as a core and hydrophilic PEG on the surface.

Both disulfiram and polymer were dissolved in acetone, which serves as the dispersed phase. Aqueous phase with or without surfactant was added to the organic phase, dropwise with constant stirring. Stirring was continued to evaporate the organic phase, followed by centrifugation at 30,000 rpm for 45 min to recover the nanoparticles. The pellet obtained was washed and resuspended in distilled water and the nanoparticles were then lyophilized for 48 h. The resulting nanoparticles were stored at 4°C.

Blank nanoparticles were formulated similarly without adding the drug. HITC-iodide, a NIR dye, loaded nanoparticles were also generated in a similar manner by the addition of dye.

### Optimization of formulation

The solvent evaporation technique (process), as well as the ingredients and their composition (formulation), has many variables, and we evaluated them keeping the low particle size, polydispersity index below 0.3 and high entrapment efficiency as the quality attributes. The process variables such as sonication, the speed of homogenization, evaporation time and centrifugation time, etc., were investigated first and the optimized parameters were used further. The formulation variables such as solvent, polymer molecular weight, surfactant, drug/polymer ratio and organic/aqueous phase ratio were taken into consideration and thoroughly investigated by changing one of them at a time while keeping the rest constant. The design of experiments and the effect of various variables on the particle size, PDI and entrapment efficiency are listed in the Supplementary Table 2.

### Measurement of particle size, size distribution, zeta potential, and morphology of mPEG-PLGA nanoparticles

The Z-Average size and zeta potential of DSF loaded mPEG-PLGA nanoparticles (DFSNPs) suspended in water were determined using dynamic light scattering (Zetasizer 3000HS, Malvern Instruments Ltd, UK). The particle size was determined using a He-Ne laser beam at a wavelength of 633 nm with a fixed scattering angle of 90° at 25°C. The data for a mean value for the size (z-Average) and a width parameter, Polydispersity Index (PDI) was evaluated using cumulants analysis. The PDI is a dimensionless, simple 2-parameter fit to the cumulants analysis and indicates the deviation of the measured autocorrelation function during the particle size estimation from that of monodisperse spheres having the same diameter. The PDI values range from 0 to 1. The zeta potential values were measured by laser Doppler velocimetry at 25°C at the default parameters of the dielectric constant, refractive index, and viscosity of water, using a disposable capillary cell with a volume of 1 mL. The morphology of the mPEG-PLGA nanoparticles was examined by transmission electron microscopy (Hitachi H-9500, Hitachi High Technologies America, Inc. Dallas, TX). Freeze-dried nanoparticles were dissolved in water with 0.05% Tween-80, and a small droplet was placed on a carbon-coated copper grid, followed by drying at room temperature before measurements were taken.

### Powder X-Ray diffraction

The samples were examined at room temperature with a Rigaku Ultima III powder diffractometer. X-ray diffraction patterns were obtained by scanning a 2θ range of 5–60°, step size = 0.02°, and a scan time of 30 sec/degree for samples PEG: PLGA- 5:45 KDa and DSF. For DSFNPs, the 2θ range and step size were kept the same with scan times increased to 60 sec/degree. The X-ray source was Cu K (alpha) radiation (*λ* =1.5418 Å) at an anode voltage of 40 kV and a current of 44 mA. The beam was then filtered by Rigaku›s Cross Beam parallel beam optics to create a monochromatic parallel beam. Data were recorded at a detector distance of 285 mm using a scintillation detector. The samples were prepared as standard powder mounts, and the diffractograms were processed through the software JADE v9.1.

### Thermal analysis

Thermal analysis was performed to complement the data generated by X-ray diffraction, to confirm the content of DSF in the formulation and to confirm the presence of disulfiram in an amorphous state in the nanoparticle matrices. DSC is used to determine the nature and speciation of crystallinity within nanoparticles through the measurement of glass and melting point temperatures and their associated enthalpies. DSC analyses were performed using a DSC822e thermal analyzer (Mettler Toledo International Inc. Columbus, OH). Samples containing approximately 5 mg of lyophilized nanoparticles were hermetically sealed in DSC aluminum pans. DSC scans were first recorded at a heating rate of 10°C min^−1^ from 0°C to 100°C and then allowed to cool to 10°C to observe the exothermic phase. During thermal scans, the crucible was purged with nitrogen. The generated data were analyzed STARe software. As a control, the pure material was analyzed to observe the change of the *T*_m_ or *T*_g_.

Thermogravimetric analysis (TGA) was used to measure the physical and chemical changes of DSFNPs that occurs as a function of temperature. The procedure measures the relative change in weight of the nanoparticles with temperature changes. A Mettler TGA/SDTA851e thermobalance was used for measurements. Approximately 5 mg of pure DSF, blank polymer or DSFNPs were heated at a flow rate of 10°C/min in an aluminum crucible from 0 to 800°C under nitrogen spurge, and the mass percentages are obtained using STARe software. The mass percentage of the sample was plotted against temperature.

### HPLC analytical method

HPLC based quantitative analysis was performed on Varian ProStar HPLC system equipped with a UV detector. Separation of analytes was carried on a Kinetex EVO C18 5 μM 100 A° column (100 × 4.6 mm, Phenomenex, CA) at room temperature. The mobile phase comprised of 70% acetonitrile and 30% water, which was pumped at a flow rate typically of 1 mL/min while the injection volume was 10 μL. The samples were measured at 254 nm wavelength. The retention time for DSF and internal standard (dimethyl thiocarbamate) was at 3.6 and 2 min respectively.

### Characterization of the drug loading and entrapment efficiency of DSFNPs

The drug loading and encapsulation efficiency of DSFNPs were determined using earlier procedures. Briefly, 1 mg of freeze-dried DSFNPs were dissolved in 1 mL of methanol and incubated for 1 h in a 37°C water bath for complete extraction of the drug. The samples were centrifuged at 13,500 rpm for 5 min, and the amount of DSF in the supernatant was determined using HPLC as described in the previous section. The drug loading and encapsulation efficiency were defined as the ratio of the amount of DSF encapsulated in nanoparticles to the total amount of DSFNPs, and the ratio of the amount of encapsulated DSF to that initially added in the process respectively. The equations are shown below.

$$\%\mathrm{LE}=\frac{\text{Drug encapsulated}}{\text{Nanoparticle weight}}*100$$

$$\%\mathrm{EE}=\frac{\text{Drug encapsulated}}{\text{Drug initially added}}*100$$

### *In vitro* release kinetics of DSFNPs

The cumulative release of DSF from the formulation was studied with different media that mimicked the physiological pH. We used simulated gastric fluid (PBS, pH 1.2), simulated intestinal fluid (PBS, pH 6.8) and simulated plasma (PBS, pH 7.4) using the dialysis method. Briefly, 10 mg of DSFNPs were dissolved in 2 mL of simulated fluid or PBS and sealed in a dialysis bag with a MW cut-off 10–13 kDa. The dialysis bags were placed in 100 mL of outer medium containing 0.05 % tween 80 at 37°C. The release medium (about 0.2 mL) was withdrawn at 15, 30, 60 min, and 1, 2, 4, 8, 12, 24, 48, 72, 96, 120, 144, 168, and 192 h. These samples were analyzed by HPLC.

### Permeability studies

The established *in vitro* model of the blood brain-barrier, IMR-90, was used for determining the permeability of DSFNPs. Cells were seeded on polycarbonate 6-well Transwell^®^ inserts with a mean pore size 3.0 μm, 0.5 mL apical volume and 1.5 mL basolateral volume, (Corning Costar Inc., NY) at a density of 5 × 10^5^ cells/well. Cells were allowed to grow at regular cell culture conditions until confluent and coherent monolayers (7–8 days) were formed; this was confirmed by measuring the transepithelial electrical resistance (TEER) of the monolayer in a growth medium at room temperature using an epithelial volt-ohm meter (EVOM, WPI Inc.). The actual TEER was calculated by subtracting the TEER value measured from a blank well without cells from the wells with cells to nullify the resistance offered by the media. The wells offering a resistance of at least 30 Ω.cm^2^ were only used for further permeability studies. The studies were carried out in Hank’s balanced salt solution (HBSS) buffer containing 30 mM HEPES at pH 6.0. Monolayers were washed with HBSS prior to the experiment, after which 0.5 and 1.2 mL of HBSS was placed into the upper and lower compartments respectively. At time zero, the DSFNPs were added to the apical chamber and were maintained under regular cell culture conditions. A total volume of 100 μL of a solution (1 or 5 μg/mL) was taken from the lower compartment at regular intervals up to 120 min and replaced with the same volume of fresh buffer, followed by the HPLC analysis. Apparent permeability coefficients ($P_{\mathrm{app}}$) were calculated by the following equation

where “∂Q/∂t” is the permeability rate of the drug across the cells, “*A*” is the diffusion area of the monolayer and “*C*_0_” is the initial concentration of DSF in the upper compartment.

### Cellular uptake of DSFNPs

The cellular internalization was quantified through an established HPLC method. Briefly, DAOY and T98G cells were incubated in a 6-well plate until they grew to 70% confluency. Next, they were treated with 40 μM of DSF or DSFNPs for 0.5, 1, and 2 h or with 10, 20, and 40 μM of DSF or DSFNPs for 1 h. Cells were washed with cold PBS and kept at −80°C for cell lysis. DSF and cellular proteins were extracted by sonication followed by centrifugation. The DSF present in the supernatant was extracted into methanol followed by HPLC for quantitation. The drug amount was normalized to the protein content in each sample. Using a NIR dye encapsulated nanoparticles we performed confocal imaging as well as flow cytometric analysis to understand the extent of uptake in a time-dependent manner. Cells were incubated with HITCNPs, a NIR dye encapsulated NPs for 30 min, 1 h and 2 h followed by washing and fixing with 4% formaldehyde. Next, the cells were analyzed by flow cytometry or counterstained with DAPI followed by imaging under a multi-photon confocal microscope (Nikon Instruments Inc).

### *In vivo* real-time imaging of NIR dye-encapsulated nanoparticles

The far-red NIR dye HITC iodide encapsulated mPEG-PLGA nanoparticles were administered to CD-1 mice at 0.2 μg of HITC/kg as a single *iv* bolus injection through the tail vein. Mice were imaged at 0 and 10 min and 1, 6, 12, and 24 h post- injection using a Calipers IVIS Lumina XR Imager (Caliper Life Sciences Inc., Hopkinton, MA). The fluorescence filters were set at ex. 710 nm and em. 800 nm. Images were taken under auto-exposure setting with high lamp intensity after the last time point, mice were euthanized followed by cervical dislocation. All vital organs were extracted, washed twice with PBS, blotted dry and transferred to a petri dish. *Ex vivo* organ fluorescence intensities were recorded at similar imaging settings. Living imaging software was used to analyze the imaging data generated. Regions of interest (ROI) were drawn into the brain/skull region of CD-1 mice, and average radiance obtained was plotted against time to get fluorescence kinetic curves of HITCNPs. Average radiance ([p/s/cm^2^/sr]/[μW/cm^2^]) refers to the sum of the radiance from each pixel inside the ROI divided by the number of pixels. Average radiance AUC values were calculated from the fluorescence kinetic curves and normalized to that of plain HITC dye.

$$\text{Apparent permeability coefficient}=\frac{\left(\partial Q/\partial t\right)}{\left(A*C_{0}\right)}$$

### Subcellular localization of DSFNPs

Approximately 5 × 10^4^ T98G glioblastoma cells were seeded on coverslips in placed in 6-well plate and were incubated at 37°C for 24 h. Then, the cells were treated for 15 min or 60 min with 100 mg/mL HITCNPs followed by the LysoTracker Red DND-99 (50 nmol/L), a marker of endolysosomal compartments or DiOC6(3) (20 nmol/L), a marker of mitochondria; the cells were further incubated for 30 min. After nuclear staining with DAPI, the cells were washed, fixed with 4% paraformaldehyde, and mounted in a fluorescent mounting medium. Images were captured with a confocal microscope and were superimposed to determine the intracellular localization of the HITCNPs.

### Western blotting

After trypsinization, the cell pellets were washed with cold TBS, and subjected to sonication in 50 mmol/L Tris–HCl (pH 8.0) containing 1% glycerol, 1 mmol/L EDTA, β-mercaptoethanol, 0.5 mmol/L PMSF and 2 mmol/L benzamidine and centrifuged. Equal protein amounts from different treatments were electrophoresed on 12% SDS–polyacrylamide gels. Proteins were electro-transferred to Immobilon-P membranes. The membranes were blocked with 5% non-fat dry milk in Tris-buffered saline (TBS; pH 8.0) containing 0.1% Tween 20 for 2 h, and subsequently incubated with appropriate primary and secondary antibodies. The antigen-antibody complexes were visualized by enhanced chemiluminescence (Pierce Company, Woburn, MA, USA). Band intensities were quantified using the ImageJ software.

### Measurement of ROS

Following treatment with DSFNPs or DMSO, T98G and DAOY cells were stained with either DHE (which is oxidized by superoxide ions into ethidium bromide and fluoresces red) or DCF-DA (which is oxidized by peroxide radicals to DCF, fluoresces green). Fluorescently labeled cells were analyzed flow cytometrically for quantification. Cells that were labeled on the coverslips were examined using fluorescence microscopy. Antimycin and hydrogen peroxide are used as positive controls for DHE and DCF-DA staining respectively, whereas N-acetyl cysteine is used as negative control in both cases.

### Analysis of ΔΨ_m_ dissipation

Approximately 5 × 10^5^ cells were plated in T-25 flasks and were incubated for 24 h followed by treatment with DSFNPs and controls. Cells were trypsinized after 24 h and were suspended in 1 mL PBS after being centrifuged and were kept on ice until staining. We followed the protocol reported by Rottenberg *et al.* [51] Briefly, cells were incubated with a lipophilic, cationic, fluorescent dye 3, 3′-Di-n-hexyloxacarbocyanine iodide (DiOC_6_(3)). DiOC_6_ (2 nM) at 37°C for 20 min, later the cells were incubated with DHE (10 μM) for 10 min. The cells were then aliquoted into two portions, CCCP (50 μM) was added to one portion, and were incubated for 20 more min. The cell fluorescence was measured by flow cytometry with appropriate channels. We used antimycin and N-acetylcysteine as positive and negative controls to the drug treatment.

### Immunofluorescence

Cells were seeded onto cover-slides the day before starting experiments. After treatment with DSFNPs or respective control compounds, cells were washed in PBS, prefixed in 4% paraformaldehyde for 15 min at 37°C and. Slides were then washed with PBS for three times before blocking for 1 h at RT with 5% BSA and 0.03% TritonX-100. Later, anti-LAMP1, 1:200 and anti-AIF 1:200 in PBS/1%BSA/0.03% Triton X-100). Slides were washed twice with PBS and incubated for 1 h at room temperature with the respective secondary antibodies at 1:1000 dilution. Images were taken under a confocal microscope Nikon A1MP at 100x objective magnification.

### Annexin V-FITC detection of apoptosis

T98G and DAOY were harvested after treatment with DSFNPs for 24 h, washed with PBS and resuspended in 0.5 mL of binding buffer (5X:10 mM HEPES pH 7.4, 150 mM NaCl, 2.5 mM CaCl_2_, 1 mM MgCl_2_, 4% BSA). The cell suspensions (500 μl) were then incubated with 5 μL of Annexin V-FITC (BD Bioscience) and 10 μL (50 μg/mL) of propidium iodide for 15 min at 37°C. The population of annexin V-positive cells was evaluated by flow cytometry [52].

### *In vitro* cytotoxicity

The DSF and DSFNPs were evaluated in parallel for assessing the cytotoxicity in six human brain cancer cell lines following a 72 h continuous drug exposure. The resazurin reduction assay was used to evaluate cell survival, where viable cells reduce resazurin to a highly fluorescent resorufin. The cell lines were seeded in 96- well microtiter plates at a density of 4000 cells/ well and were incubated for 24 h before drug additions. Next, DSF and DSFNPs at ten serially diluted concentrations were added in triplicate for a further 72 h. Next, 20 μL resazurin 0.01% (w/v) was added to each well. After 2 h, the fluorescence was measured using a Synergy 2 multi-mode Reader (BioTek Instruments Inc.) at a 530-nm excitation and 590-nm emission. Cell viability was expressed as a percentage of the fluorescence to that of the untreated controls, and the fluorescence of blank wells (without cells) was subtracted.

### Clonogenic survival assays

A total of 400 cells/well T98G and DAOY cells were seeded in a 6-well plate, and plates were incubated for 24 h followed by addition of IC_20_ and IC_40_ concentrations of DSF and DSFNPs. After 24 h, fresh media was added, and plates were incubated for 2 weeks. The cells were next washed with PBS and fixed with methanol at -20 ^0^C for 15 min followed by staining with 0.5% crystal violet for 20 min. Colonies were counted using the ImageJ software.

### Development of orthotopic medulloblastoma xenografts and drug efficacy studies

As the first step, the human medulloblastoma DAOY cells were engineered to express luciferase by stable transfection using the RediFect Red-Fluc-Puromycin lentiviral particles (Perkin Elmer) according to the manufacturer’s instructions. Polybrene was used to enhance the transfection efficiency. The selection was performed in the presence of 1 mg/ml of Puromycin. Stable transfection was confirmed by luciferase activity measurements in cell lysates and imaging the cells in the presence of luciferin in IVIS.

Four-week-old male and female NCG mice, which lack T, B, and natural killer cells (triple immune-deficient) were housed in a micro ventilated caging system in a sterile environment and fed *ad-libitum* with standard irradiated research rodent diet and water. The animals were anesthetized using 2% isoflurane and positioned in a Benchmark (Leica) stereotactic instrument. A 27-gauge needle was then used to drill a burr hole into the skull 0.5-mm anterior and 2-mm lateral to the bregma. DAOY-Luc2 cell suspension (2 × 10^5^ cells in 5 μl PBS) was injected in the striatum at a depth of 5 mm from the dural surface over 10 minutes. These mice were imaged for bioluminescence five days post tumor inoculation and were observed for stable tumor growth for the next two weeks.

The non-invasive *in vivo* bioluminescence measurements were performed using an IVIS-200 Caliper Imaging System. The animals lying in the prone position were given i.p. injections of D-luciferin (2 mg in 100 µl PBS per mouse) followed by anesthesia. Imaging was performed exactly 10 min after the luciferin injections. Measurements of signal intensity were obtained from a region of interest analysis encompassing the intracranial area using the Living Image software. Bioluminescence was expressed as a total radiance in photons per sec/cm^2^ per steradian. The linearity of photon emission in our setting was optimized using a previous procedure [53].

Mice with stable and equivalent intracranial tumors were then randomly divided into four groups with 6 mice in each group, namely the vehicle controls, DSF, blank NPs, and DSFNPs. Power analysis was used to calculate that a minimum of 6 mice/group will be required to detect differences between tumor volumes assuming an 80% power, the desired P value of 0.05, and a common standard deviation of 2.0. DSF or DSFNPs were given by i.p. injections at 30 mg/kg three times a week. Tumor growth of all mice was monitored once every week 10 minutes after an *i.p.* injections of D-luciferin as described above. Mice were monitored periodically for clinical signs of tumor burden and body weights tracked.

### Statistical analysis

The data are expressed as the means ± SEM from at least three independent experiments. Two-sided *t*-tests were used for comparisons between two groups. A value of *P* < 0.05 was statistically significant at 95% confidence interval. 1way ANOVA with Dunnett multiple comparisons test was performed for *in vivo* tumor efficacy studies.

## SUPPLEMENTARY MATERIALS FIGURES AND TABLES



## Abbreviations

PdI: Polydispersity Index
DLS: Dynamic light scattering
TEM: Transmission electron microscopy
SEM: Scanning electron microscopy
XRD: X-ray diffraction
DSC: Differential scanning calorimetry
TGA: Thermo gravimetric analysis
PBS: phosphate-buffered saline
FBS: fetal bovine serum
FACS: fluorescence-activated cell sorting
HITC: the near-infrared dye 1,1′,3,3,3′,3′- Hexamethyl indotricarbocyanine iodide
HITCNPs: HITC encapsulated nanoparticles
DSF: Disulfiram
DSFNPs: DSF encapsulated nanoparticles
MDC: Mono dansyl cadaverine
LAMP1: Lysosome-associated membrane glycoprotein 1
FITC: Fluorescein isothiocyanate
PE: Phycoerythrin
DCF-DA: 2′,7′–Dichlorofluorescin diacetate
DHE: Dihydroethidium
CCCP: Carbonyl cyanide m-chlorophenyl hydrazine
DiOC6(3): 3,3′-Dihexyloxacarbocyanine iodide
NAC: N-acetylcysteine
MAPK: Mitogen-activated protein kinase
AIF: Apoptosis inducing factor
CHOP: Transcription factor C/EBP homologous *protein*
JNK: c-Jun N-terminal kinase
ERK: Extracellular signal-regulated kinase
ACN: Acetonitrile
DCM: Dichloromethane
EA: Ethyl acetate
PVA: Polyvinylalcohol
mPEG: methoxy polyethylene glycol
PLGA: Poly lactic-*co*-glycolic acid
ROS: Reactive oxygen species
MGMT: O6-Methylguanine DNA methyltransferase
BBB: Blood Brain-barrier
i.p.: intraperitoneal
