# Supplementary material for: Brain- and brain tumor-penetrating disulfiram nanoparticles: Sequence of cytotoxic events and efficacy in human glioma cell lines and intracranial xenografts
Source: Oncotarget. 2017 Dec 15;9(3):3459–82. doi: 10.18632/oncotarget.23320 (PMC5790476; doi:10.18632/oncotarget.23320)
Supplement: Supplementary file 1 [file oncotarget-09-3459-s001.pdf]

Brain- and brain tumor-penetrating disulfiram nanoparticles: Sequence of cytotoxic events and efficacy in human glioma cell lines and intracranial xenografts

SUPPLEMENTARY MATERIALS

DSF-nanoparticles: evaluation of process and formulation variables

One of the most successful and reproducible methods used for NP preparation of hydrophobic drugs is the single emulsion solvent evaporation technique [1]. We improved the formulation by altering the process and formulation variables and used particle size below 100 nm, PDI below 0.1 and more than 90% encapsulation efficiency as end points. The process variables such as homogenization (speed and time), evaporation time and centrifugation (speed and time) were considered. We observed 8 h of stirring was enough to facilitate the complete evaporation of solvent and centrifuging the formulations for 30 mins with 18,000 rpm yielded a uniform formulation. Homogenization resulted in homogenous particles with a low particle diameter in the case of solvents such as DCM and ethyl acetate though, we did not notice much difference in the case of acetone (data not shown). Later, we further improved our formulation by changing the variables such as solvent type, drug/ polymer ratio, organic/aqueous phase ratio, surfactant, and polymer molecular weight.

Influence of type of solvent

We chose a range of water miscible [acetone, acetonitrile (ACN), dimethylformamide (DMF), tetrahydrofuran (THF)]; partially miscible [ethyl acetate (EA), propylene carbonate (PC)] and immiscible [carbon tetrachloride (CTL), chloroform, dichloromethane (DCM)] solvents to test the effect of different types of solvents on particle size, PDI and drug entrapment efficiency (% EE). The water-soluble solvents acetone and acetonitrile formed a homogenous formulation with the particle size and PDI of 78, 141.3 nm and 0.09, 0.04 respectively and the % EE were 92.7 and 85.3%, whereas THF formed a formulation with two populations of particles with different sizes with an average size of 94.6 nm, PDI of 0.39 and % EE of 82.4 %. DMF formed a viscous formulation and particle size was 765 nm with a 0.61 PDI and EE of 50.2%. Use of partially miscible solvents such as EA and PC resulted in a formulation with 510.2, 653.1 nm size and 0.23, 0.24 PDI with 42.8 and 47 % EE respectively. Water immiscible solvents such as chloroform, DCM and CTL resulted in a formulation with large particle size with a z-average size of 418, 304.4, 553 nm; 0.41, 0.39, 0.45 PDI and 66, 82, 56 % EE respectively (Supplementary Figure 1A, Supplementary Table 2). From all the solvents that we examined, we chose acetone, ACN, THF, DCM, and EA for further optimization and Supplementary Figure 1B represent the size distribution curves the formulations made with these solvents.

Influence of organic/aqueous phase ratio

The particle size decreased with increase in the dispersion phase composition. We examined 1:5, 1:10, 1:15 and 1:20 ratios of organic/ aqueous ratio with acetone, ACN, and DCM as organic phase. As shown in the Supplementary Figure 2A (a-c) we observed a clear decrease in particle size at 1:10 organic/ aqueous phase ratio compared to rest of the formulations. The % EE ranged from 68 to 92% with all three organic solvents at 1:10 proportion of organic to aqueous phase and we did not observe much change in encapsulation efficiency from 1:10 to 1:20 ratios. These findings can be explained on the basis of change in viscosity of emulsion associated with change in the proportion of organic and aqueous phases as viscosity plays a key role in imposing resistance to shear force during the formation of nanoparticles [2] and further increase in aqueous phase might have dissolved a significant amount of drug in it causing drug loss [3] leading to non-homogeneous formulation as observed with increase in PDI and particles being in two different populations causing an overall increase in particle size. To sum it up, we opted to continue further evaluations with organic/ aqueous phase ratio of 1:10.

Effect of drug/polymer ratio

We chose formulation 1 to evaluate the effect of increasing polymer composition compared to drug revealed that increase in drug to polymer ratio has positive impact on the quality attributes in question. We observed decrease in z-average size from 385 nm to 85 nm and PDI from 0.42 to 0.18 with an increase in %EE from 80 to 96 (Supplementary Figure 2B), probably for the

reason that increased polymer increases the viscosity in aqueous phase and thereby stabilizes the nanoparticles and prevents the outflow of drug which ultimately produces compact nanoparticles with a better entrapment [4]. Much increase in viscosity as observed in same formulation with surfactants such as polyvinyl alcohol (PVA) and Pluronic® F68 resulted in larger particle size with an improved entrapment though. The reason could be that much increase viscosity hinders effective diffusion of organic phase and may impose resistance to shear force leading to larger size of nanoparticles [2, 5].

Influence of surfactants

The effect of presence of surfactant and its concentration on particle size and PDI is studied using tween 80, poloxamer 188 (Pluronic® F68), polyvinyl alcohol (PVA) and poloxamer 407 (Pluronic® F127) in formulation 1. The z-average size of the nanoparticles increased with the increasing concentration of tween 80, Pluronic F68 and PVA where as we observed decrease in size with Pluronic® F127 across 0.5 to 2 %w/v but it was still above 100 nm and the PDI values were irregular, so we decided not to use any surfactant in our final formulation (Supplementary Figure 2C (a-d)). But with rest of the solvents we examined the effect of presence of Pluronic® F68 in coming studies.

Influence of solvent vs surfactant vs polymer molecular weight

Supplementary Figure 3 reveals results of further optimization of DSFNP formulation, where three variables, polymer molecular weight, solvent type, and surfactant with particle size, PDI and %EE as the end points. The results are also summarized in Supplementary Table 2. Increase in polymer molecular weight was associated with an increase in chain length. Use of Pluronic® F68 helped to get a uniform formulation with relatively less particle size in the formulations made from EA. Whereas, the particle size increased with its use in formulations made from acetone or acetonitrile irrespective of the polymer molecular weight. Moreover, the particle size and %EE of the formulation made with acetone without surfactant was much better compared to rest of the solvents. Regarding the influence of polymer molecular weight or polymer chain length, with a constant mPEG (3 kDa) chain length, we found the particle size to increase with increase in chain length of PLGA (7.5, 19, and 30 kDa), which can be attributed to the increase in the viscosity of organic phase and increase in the size of PEG brush. We did not observe much increase in the particle size with increase in PEG from 3 to 5 kDa, though it resulted with significantly higher %EE. Altogether, among the five polymers tested, we observed a lower particle size, PDI and higher %EE in the formulation made with mPEG (5 kDa): PLGA (45 kDa) using acetone as organic phase without surfactant (Supplementary Table 2). Our choice of the polymer is further strengthened by the literature, higher PLA/PEG ratio forms more solid like nanosphere structure [6]; higher PLA though results in higher core radius, though it affects the conformation of PEG chains in the shell leading to decreased PEG chain grafting density, which further makes the core more radially homogenous [7]; the lower PEG content leads to lower stability of formulation for its possible incomplete coverage, which further may exhibit higher aggregation during storage [8]; PEG 5 kDa or higher MW is usually suggested as distance between two PEG molecules should be less than 1.4 nm in order to avoid opsonization of NPs by reticuloendothelial cells [9]; long chains of PEG are recommended to limit the protein adsorption [10].

To summarize, we enhanced the formulation characteristics in terms of the particle size, loading and encapsulation efficiencies by adjusting the method variables like solvent, polymer, their composition, and the procedures involved. Among the different formulations, we tried, the formulation 1, having optimum parameters, was further characterized. The final recipe of our formulation is, DSF and polymer in 1:10 are dissolved in 1 mL of acetone followed by addition of distilled water slowly drop by drop with continuous stirring for 8 h. Then the resultant formulation was washed thrice and was centrifuged. Further the pellet was re-dispersed in distilled water. Water and was freeze dried to obtain the powdered nanoparticles which were further characterized and used for *in vitro* and *in vivo* experiments.

REFERENCES

1. Zhang Y, Chan HF, Leong KW. Advanced Materials and Processing for Drug Delivery: The Past and the Future. Advanced drug delivery reviews. 2013; 65:104–20. https://doi.org/10.1016/j.addr.2012.10.003.
2. Quintanar-Guerrero D, Fessi H, Allémann E, Doelker E. Influence of stabilizing agents and preparative variables on the formation of poly(d,l-lactic acid) nanoparticles by an emulsification-diffusion technique. International Journal of Pharmaceutics. 1996; 143:133–41.
3. Sharma N, Madan P, Lin S. Effect of process and formulation variables on the preparation of parenteral paclitaxel-loaded biodegradable polymeric nanoparticles: A co-surfactant study. Asian Journal of Pharmaceutical Sciences. 2016; 11:404–16.
4. Bhadra S, Prajapati AB, Bhadra D. Development of pH sensitive polymeric nanoparticles of erythromycin stearate. Journal of Pharmacy & Bioallied Sciences. 2016; 8:135–40. https://doi.org/10.4103/0975-7406.171691.
5. Guhagarkar SA, Malshe VC, Devarajan PV. Nanoparticles of Polyethylene Sebacate: A New Biodegradable Polymer. AAPS PharmSciTech. 2009; 10:935. https://doi.org/10.1208/s12249-009-9284-4.
6. Stolnik S, Heald CR, Neal J, Garnett MC, Davis SS, Illum L, Purkis SC, Barlow RJ, Gellert PR. Polylactide-poly(ethylene glycol) micellar-like particles as potential

drug carriers: production, colloidal properties and biological performance. J Drug Target. 2001; 9:361–78.

7. Avgoustakis K. Pegylated poly(lactide) and poly(lactide-co-glycolide) nanoparticles: preparation, properties and possible applications in drug delivery. Curr Drug Deliv. 2004; 1:321–33.
8. Avgoustakis K, Beletsi A, Panagi Z, Klepetsanis P, Livaniou E, Evangelatos G, Ithakissios DS. Effect of copolymer composition on the physicochemical characteristics, *in vitro* stability, and biodistribution of PLGA-mPEG nanoparticles. Int J Pharm. 2003; 259:115–27.
9. Vittaz M, Bazile D, Spenlehauer G, Verrecchia T, Veillard M, Puisieux F, Labarre D. Effect of PEO surface density on long-circulating PLA-PEO nanoparticles which are very low complement activators. Biomaterials. 1996; 17:1575–81.
10. Jeon SI, Lee JH, Andrade JD, De Gennes PG. Protein—surface interactions in the presence of polyethylene oxide. Journal of Colloid and Interface Science. 1991; 142:149–58.

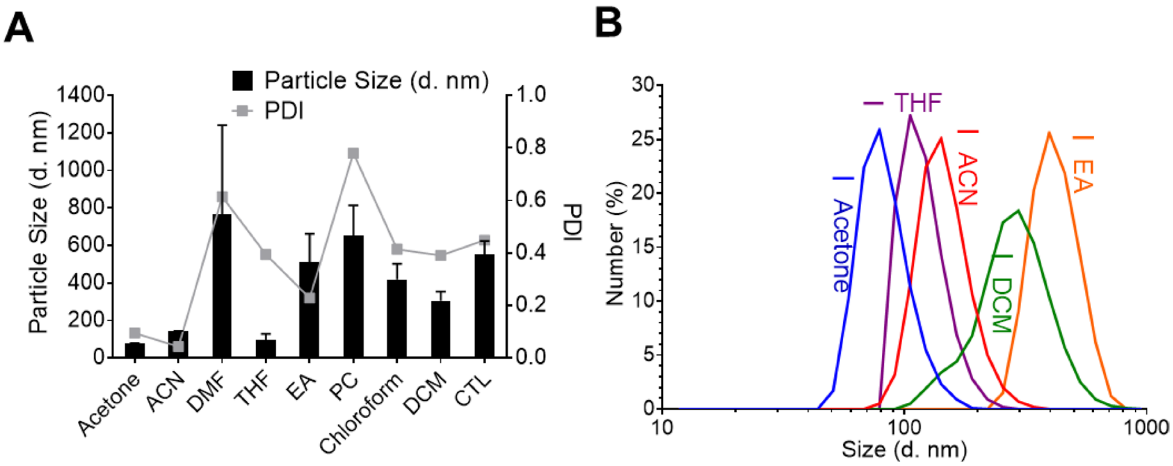

Supplementary Figure 1: Effect of miscibility of solvents. (**A**) particle size (left) and PdI (right) of formulations made from various organic solvents (**B**) size distribution curves of formulations that are made from solvents such as acetone, ACN, DCM, and EA.

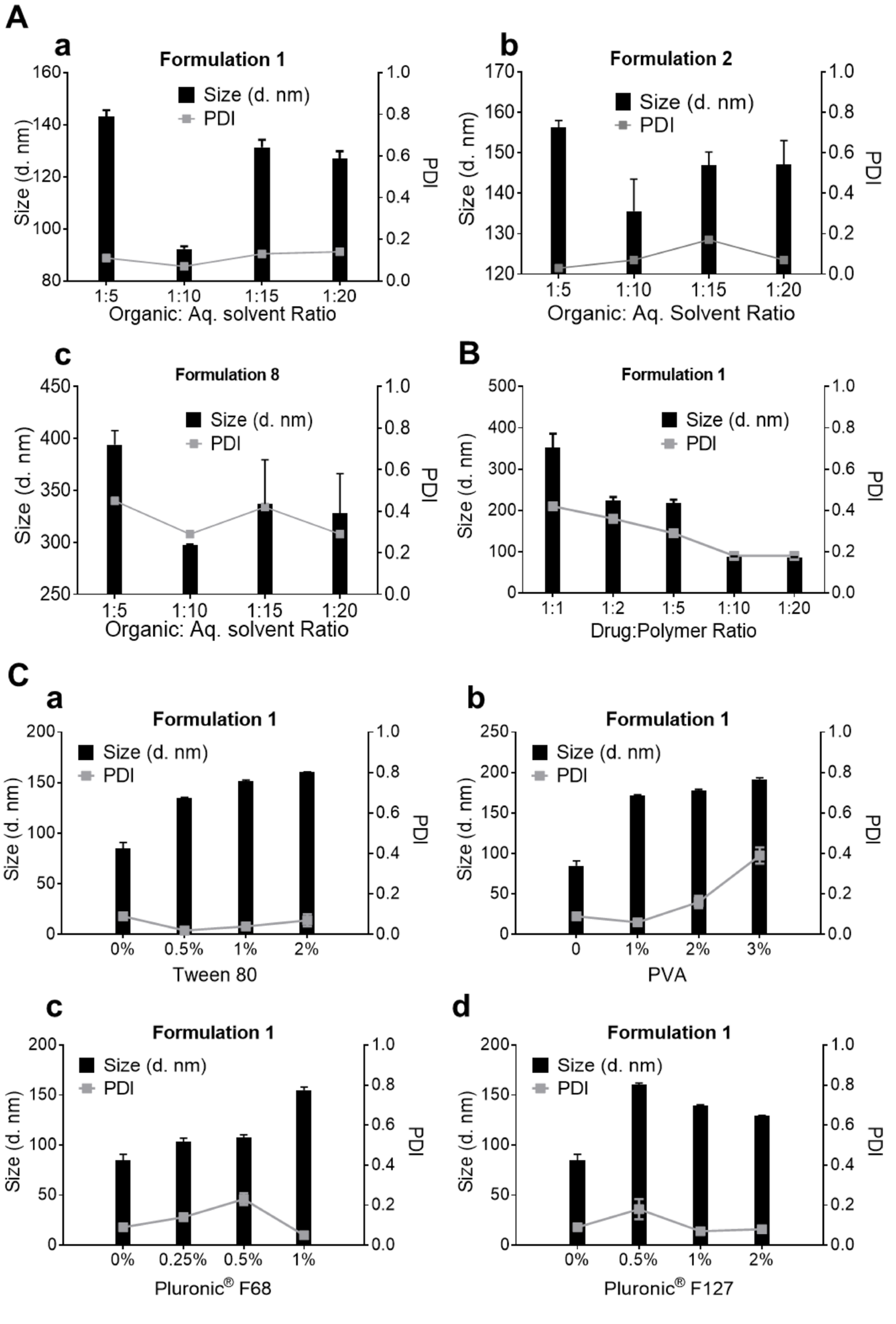

Supplementary Figure 2: Effect of formulation variables on size and PdI of DSFNPs. (**A**) Effect of the organic/aqueous phase ratio on particle size (left Y-axis) and PdI (right Y-axis) of formulation 1 (a); 2 (b); and 3 (c). (**B**) Effect of the drug/polymer ratio on particle size (left Y-axis) and PdI (right Y-axis) of formulation 1 (**C**) Effect of surfactant concentration tween-80 (a); PVA (b); Pluronic® F68 (c); and Pluronic® F127 (d); on particle size (left Y-axis) and PdI (right Y-axis) of formulation 1.

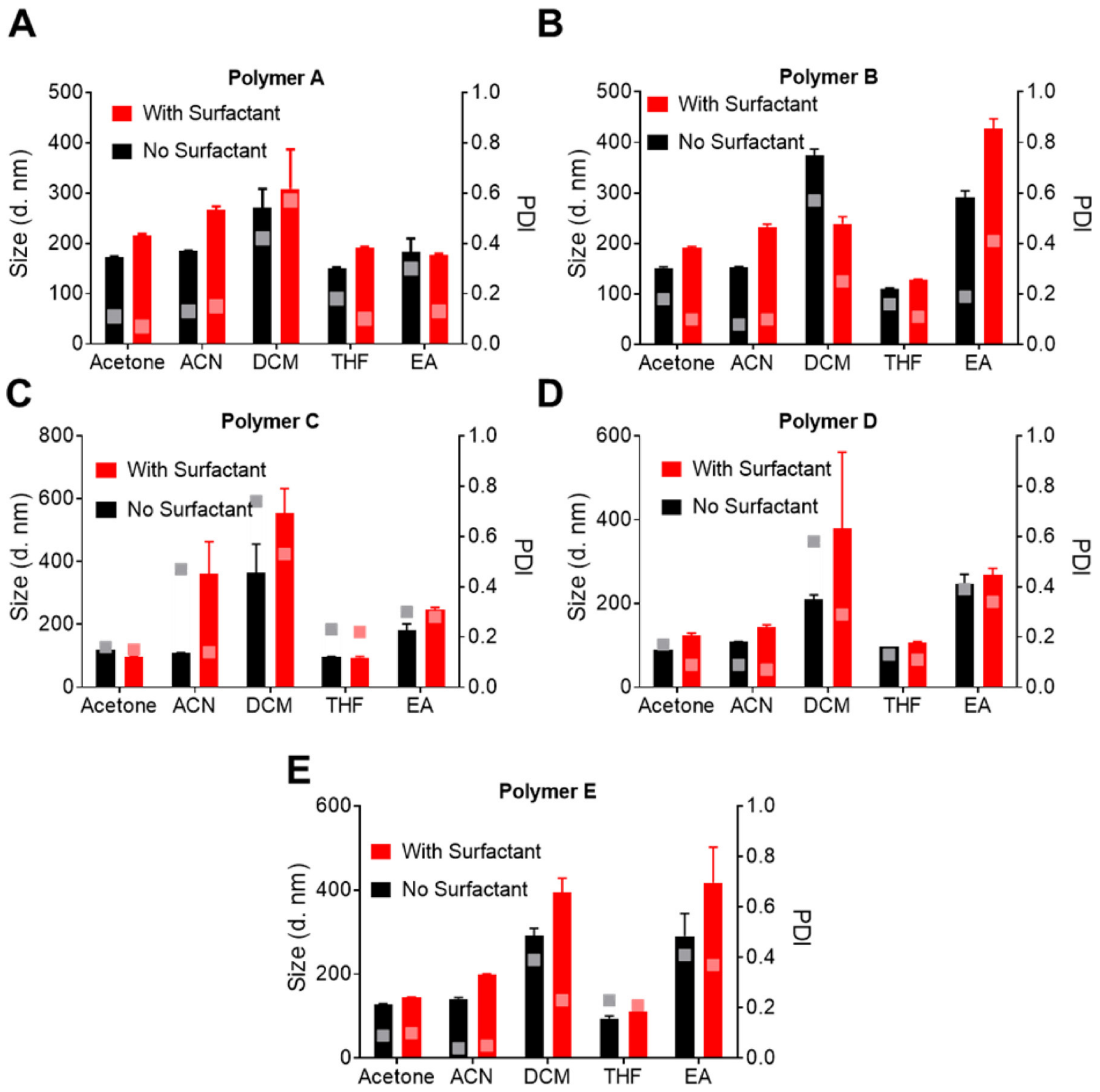

Supplementary Figure 3: Effect of formulation variables on size and Pdl of DSFNPs. Effect of solvent miscibility, surfactant presence (Red) and absence (Black) and molecular weight of mPEG: PLGA (**A**) 3:7.5 kDa; (**B**) 3:19 kDa; (**C**) 3:30 kDa; (**D**) 5:45 kDa and (**E**) 5:49 kDa on particle size (left Y-axis) and Pdl (right Y-axis) of DSFNPs.

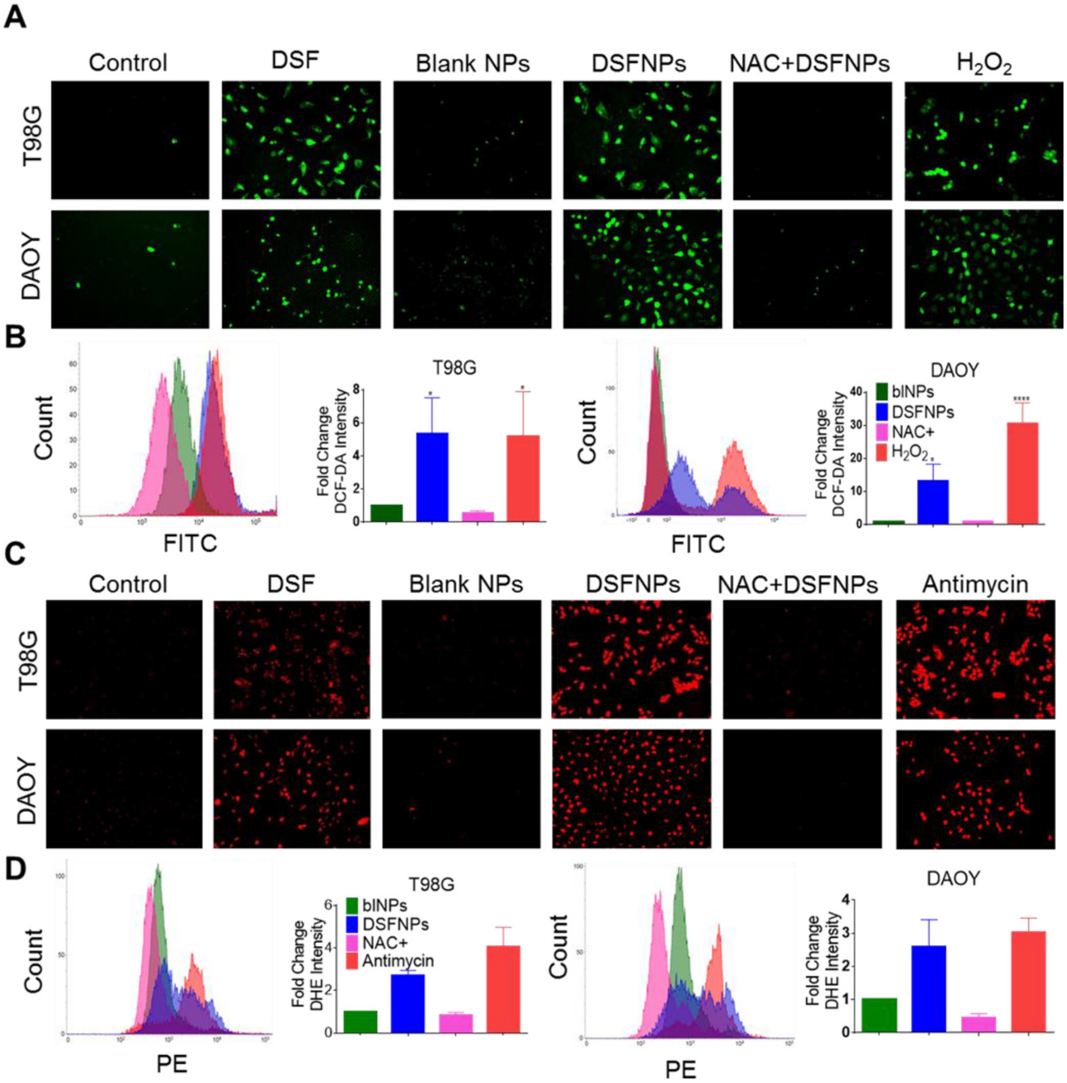

Supplementary Figure 4: DSFNPs induce release of ROS. Enhanced ROS accumulation in T98G and DAOY cells with DSF and DSFNPs alone or pre-treatment with NAC as described in materials and methods. Upon treatment cells were stained with DCF-DA (Green) and DHE (Red) to show the induction of peroxides and superoxides respectively using both fluorescence microscopy (**A**, **C**) and flow cytometry (**B**, **D**).

Supplementary Table 1: Effect of miscibility of organic solvent on particle size, Pdl and %EE of DSFNPs

Name	Solvent	Polymer	Drug:Polymer	Org:Aq Phase	Surfactant	**Size (d. nm)**	Std. Dev	**PDI**	Std. Dev	**% EE**
Formulation 1	Acetone	PEG-PLGA 5:49 kDa	1:10	1:10	No	**78.2**	6.0	**0.09**	0.002	**92.7**
Formulation 2	Acetonitrile					**141.3**	3.1	**0.04**	0.044	**85.3**
Formulation 3	DMF					**764.8**	476.0	**0.61**	0.506	**50.2**
Formulation 4	THF					**94.6**	5.8	**0.39**	0.044	**73.3**
Formulation 5	Ethyl acetate					**510.2**	5.6	**0.23**	0.016	**42.8**
Formulation 6	PC					**653.1**	49.8	**0.78**	0.079	**47.2**
Formulation 7	Chloroform					**417.7**	84.3	**0.41**	0.216	**66.5**
Formulation 8	DCM					**304.4**	34.1	**0.39**	0.102	**82.4**
Formulation 9	Carbon tetrachloride					**553.3**	70.6	**0.45**	0.066	**55.8**

Supplementary Table 2: Effect of polymer molecular weight, surfactant on particle size, Pdl and %EE of DSFNPs

S. No	Polymer	Solvent	Drug: Polymer	Org: Aq Phase	Surfactant	Size (d. nm)	SD	PDI	SD	% EE
1	PEG-PLGA 3:7.5 kDa	Acetone	1:10	1:10	No	92.3	0.7	0.11	0.01	61.9
2			1:10	1:10	Yes	116.6	2.2	0.07	0.03	66.2
3		ACN	1:10	1:10	No	184.7	1.0	0.13	0.02	58.7
4			1:10	1:10	Yes	266.5	6.8	0.15	0.01	53.6
5		DCM	1:10	1:10	No	270.1	38.4	0.42	0.08	45.2
6			1:10	1:10	Yes	308.4	78.6	0.57	0.18	46.6
7		THF	1:10	1:10	No	150.8	1.6	0.18	0.02	51.6
8			1:10	1:10	Yes	191.3	2.0	0.10	0.02	50.9
9		Et. Acetate	1:10	1:10	No	183.4	25.9	0.30	0.14	43.3
10			1:10	1:10	Yes	177.1	2.4	0.13	0.01	48.3
11	PEG-PLGA 3:19 kDa	Acetone	1:10	1:10	No	101.7	2.3	0.18	0.05	65.4
12			1:10	1:10	Yes	191.9	2.1	0.10	0.02	70.3
13		ACN	1:10	1:10	No	152.2	2.2	0.08	0.02	56.8
14			1:10	1:10	Yes	232.6	5.6	0.10	0.01	51.6
15		DCM	1:10	1:10	No	374.5	12.3	0.57	0.05	41.0
16			1:10	1:10	Yes	239.1	13.9	0.25	0.06	37.5
17		THF	1:10	1:10	No	110.7	1.5	0.16	0.04	56.9
18			1:10	1:10	Yes	128.2	1.3	0.11	0.01	73.7
19		Et. Acetate	1:10	1:10	No	290.5	14.0	0.19	0.10	61.8
20			1:10	1:10	Yes	427.6	18.9	0.41	0.13	49.2
21	PEG-PLGA 3:30 kDa	Acetone	1:10	1:10	No	120.5	3.0	0.16	0.02	71.7
22			1:10	1:10	Yes	97.1	0.9	0.15	0.03	71.3
23		ACN	1:10	1:10	No	108.9	1.8	0.47	0.21	58.2
24			1:10	1:10	Yes	360.0	103.5	0.14	0.01	69.1
25		DCM	1:10	1:10	No	364.7	90.8	0.74	0.09	43.2
26			1:10	1:10	Yes	553.3	78.9	0.53	0.18	19.3
27		THF	1:10	1:10	No	95.8	2.0	0.23	0.06	54.2
28			1:10	1:10	Yes	94.4	3.5	0.22	0.02	57.8
29		Et. Acetate	1:10	1:10	No	181.8	9.8	0.30	0.05	40.1
30			1:10	1:10	Yes	247.5	6.4	0.28	0.03	44.4
31	PEG-PLGA 5:45 kDa	Acetone	1:10	1:10	No	82.4	5.8	0.02	0.04	96.6
32			1:10	1:10	Yes	124.3	5.0	0.09	0.05	63.8
33		ACN	1:10	1:10	No	108.4	0.5	0.09	0.02	58.7
34			1:10	1:10	Yes	142.8	6.3	0.07	0.03	67.7
35		DCM	1:10	1:10	No	208.7	11.9	0.58	0.27	67.6
36			1:10	1:10	Yes	378.1	183.5	0.29	0.05	59.1
37		THF	1:10	1:10	No	97.1	0.1	0.13	0.02	89.9
38			1:10	1:10	Yes	106.9	2.3	0.11	0.03	83.9
39		Et. Acetate	1:10	1:10	No	247.3	22.5	0.39	0.06	42.3
40			1:10	1:10	Yes	268.0	16.1	0.34	0.14	55.2
41	PEG-PLGA 5:49 kDa	Acetone	1:10	1:10	No	89.6	3.2	0.17	0.00	76.8
42			1:10	1:10	Yes	129.0	0.9	0.10	0.03	98.0
43		ACN	1:10	1:10	No	141.3	3.1	0.04	0.04	79.9
44			1:10	1:10	Yes	199.5	1.5	0.05	0.03	89.9
45		DCM	1:10	1:10	No	394.4	34.1	0.39	0.10	40.5
46			1:10	1:10	Yes	291.3	17.8	0.23	0.06	36.9
47		THF	1:10	1:10	No	94.6	5.8	0.23	0.04	38.4
48			1:10	1:10	Yes	111.3	0.7	0.21	0.02	43.2
49		Et. Acetate	1:10	1:10	No	417.7	84.3	0.41	0.22	52.2
50			1:10	1:10	Yes	290.5	53.9	0.37	0.08	54.3
